# Supplementary material for: New Coumarin Dipicolinate Europium Complexes with a Rich Chemical Speciation and Tunable Luminescence
Source: Molecules. 2021 Feb 26;26(5):1265. doi: 10.3390/molecules26051265 (PMC7956443; doi:10.3390/molecules26051265)
Supplement: Supplementary file 1 [file molecules-26-01265-s001.pdf]

# SUPPORTING INFORMATION FOR

# New Coumarin Dipicolinate Europium Complexes with a Rich Chemical Speciation and Tunable Luminescence

Figure S 1. $^{1}$H-NMR spectrum of compound 1. .......... 4

Figure S 2. $^{1}$H-NMR spectrum of compound 2. .......... 5

Figure S 3. $^{13}$C-NMR spectrum of compound 2. .......... 6

Figure S 4. $^{1}$H-NMR spectrum of compound 3. .......... 7

Figure S 5. $^{13}$C-NMR spectrum of compound 3. .......... 8

Figure S 6. $^{1}$H-NMR spectrum of compound 4. .......... 9

Figure S 7. $^{13}$C-NMR spectrum of compound 4. .......... 10

Figure S 8. $^{1}$H-NMR spectrum of compound 5. .......... 11

Figure S 9. $^{13}$C-NMR spectrum of compound 5. .......... 12

Figure S 10. $^{13}$C-NMR spectrum of the triethylammonium salt of $HL_1$. .......... 13

Figure S 11. HMRS spectrum of compound $HL_1$. .......... 14

Figure S 12. HPLC chromatogram of the triethylammonium salt of $HL_1$. .......... 15

Figure S 13. $^{1}$H-NMR spectrum of compound $HL_2$. .......... 16

Figure S 14. $^{13}$C-NMR spectrum of compound $HL_2$. .......... 17

Figure S 15. HMRS spectrum of compound $HL_2$. .......... 18

Figure S 16. HPLC chromatogram of the triethylammonium salt of $HL_2$. .......... 19

Figure S 17. HRMS spectrum of $Na_3[Eu(L_1)_3]$ complex (above) with the relative calculated one (below). .......... 20

Figure S 18. HPLC chromatogram of $Na_3[Eu(L_1)_3]$ complex. .......... 21

Figure S 19. $^{1}$H-NMR spectrum of $Na_3[Eu(L2)_3]$ complex. .......... 22

Figure S 20. HRMS spectrum of $Na_3[Eu(L_2)_3]$ complex (above) with the relative calculated one (below). .......... 23

Figure S 21. HPLC chromatogram of $Na_3[Eu(L_2)_3]$ complex. .................................................... 24

Figure S 22. $^1$H-NMR titration (400 MHz, $D_2O$) of $L_1$ (triethylammonium salt) with $Eu^{3+}$ (chloride hexahydrate salt)............................................................................................ 25

Figure S 23. $^1$H-NMR titration (400 MHz, DMSOd) of $L_1$ (triethylammonium salt) with $Eu^{3+}$ (chloride hexahydrate salt), particular of the counterion dynamic. ............................................ 25

Figure S 24. Fluorescence titration spectra of $L_1^{2-}$ in Tris HCl with $Eu^{3+}$ (above) and titration curve monitoring the fluorescence at 615 nm (below). ............................................................ 26

Figure S 25. Fluorescence decay fitting of $L_1^{2-}$ in Tris HCl with increasing amounts of $Eu^{3+}$ monitoring at 615 nm and exciting at 360 nm. ............................................................................ 28

Figure S 26. Fluorescence decay fitting of $L_1^{2-}$ in Tris HCl with increasing amounts of $Eu^{3+}$ monitoring at 615 nm and exciting at 360 nm: 0.05 eq ................................................................ 30

Figure S 27. Fluorescence decay fitting of $L_1^{2-}$ in Tris HCl with increasing amounts of $Eu^{3+}$ monitoring at 615 nm and exciting at 360 nm: 0.1 eq .................................................................. 32

Figure S 28. Fluorescence decay fitting of $L_1^{2-}$ in Tris HCl with increasing amounts of $Eu^{3+}$ monitoring at 615 nm and exciting at 360 nm: 0.15 eq ................................................................ 34

Figure S 29. Fluorescence decay fitting of $L_1^{2-}$ in Tris HCl with increasing amounts of $Eu^{3+}$ monitoring at 615 nm and exciting at 360 nm: 0.2 eq .................................................................. 36

Figure S 30. Fluorescence decay fitting of $L_1^{2-}$ in Tris HCl with increasing amounts of $Eu^{3+}$ monitoring at 615 nm and exciting at 360 nm: 0.25 eq ................................................................ 38

Figure S 31. Fluorescence decay fitting of $L_1^{2-}$ in Tris HCl with increasing amounts of $Eu^{3+}$ monitoring at 615 nm and exciting at 360 nm: 0.35 eq ................................................................ 40

Figure S 32. Fluorescence decay fitting of $L_1^{2-}$ in Tris HCl with increasing amounts of $Eu^{3+}$ monitoring at 615 nm and exciting at 360 nm: 0.4 eq .................................................................. 42

Figure S 33. Fluorescence decay fitting of $L_1^{2-}$ in Tris HCl with increasing amounts of $Eu^{3+}$ monitoring at 615 nm and exciting at 360 nm: 0.45 eq ................................................................ 44

Figure S 34. Fluorescence decay fitting of $L_1^{2-}$ in Tris HCl with increasing amounts of $Eu^{3+}$ monitoring at 615 nm and exciting at 360 nm: 0.5 eq .................................................................. 46

Figure S 35. Fluorescence decay fitting of $L_1^{2-}$ in Tris HCl with increasing amounts of $Eu^{3+}$ monitoring at 615 nm and exciting at 360 nm: 0.6 eq ................................................................ 48

Figure S 36. Fluorescence decay fitting of $L_1^{2-}$ in Tris HCl with increasing amounts of $Eu^{3+}$ monitoring at 615 nm and exciting at 360 nm: 0.7 eq ................................................................ 50

Figure S 37. Fluorescence decay fitting of $L_1^{2-}$ in Tris HCl with increasing amounts of $Eu^{3+}$ monitoring at 615 nm and exciting at 360 nm: 2 eq .................................................................... 52

Figure S 38. Fluorescence spectra comparison (excitation at 360 nm) in DMSO between Eu3+/$L_2^{2-}$ in 1:2 (black), 1:3 (blue) and 1:4 (red) molar ratios................................................... 53

Figure S 39. HRMS spectra of DMSO mixture $Eu^{3+}$:$L_1^{2-}$ in 1:2 molar ratio............................... 55

Figure S 40. HMRS spectrum of DMSO mixture $Eu^{3+}$:$L_2^{2-}$ in 1:2 molar ratio. ........................... 56

Figure S 41. HMRS spectrum of DMSO mixture $Eu^{3+}$:$L_1^{2-}$ in 1:4 molar ratio. ........................... 57

Figure S 42. Fluorescence decay fitting of isolated $Na_3[Eu(L_1)_3]$ in water, monitoring at 615 nm and exciting at 360 nm. ............................................................................................ 59

Figure S 43. Fluorescence decay fitting of isolated $Na_3[Eu(L_2)_3]$ in water, monitoring at 615 nm and exciting at 360 nm. ............................................................................................ 61

Figure S 44. Phosphorescence spectrum of $Na_3[Eu(L_1)_3]$ complex in water with 200µs of delay time. ........................................................................................................................ 62

Figure S 45. Quantum yields plot of isolated tris complexes in water solution (above) and fitting details (below)........................................................................................................... 63

Figure S 46. Quantum yields plot of isolated tris complexes in DMSO solution (above) and fitting details (below). .............................................................................................. 64

Figure S 47. Fitting details of quinine sulfate in 0.5 M $H_2SO_4$. ................................................. 65

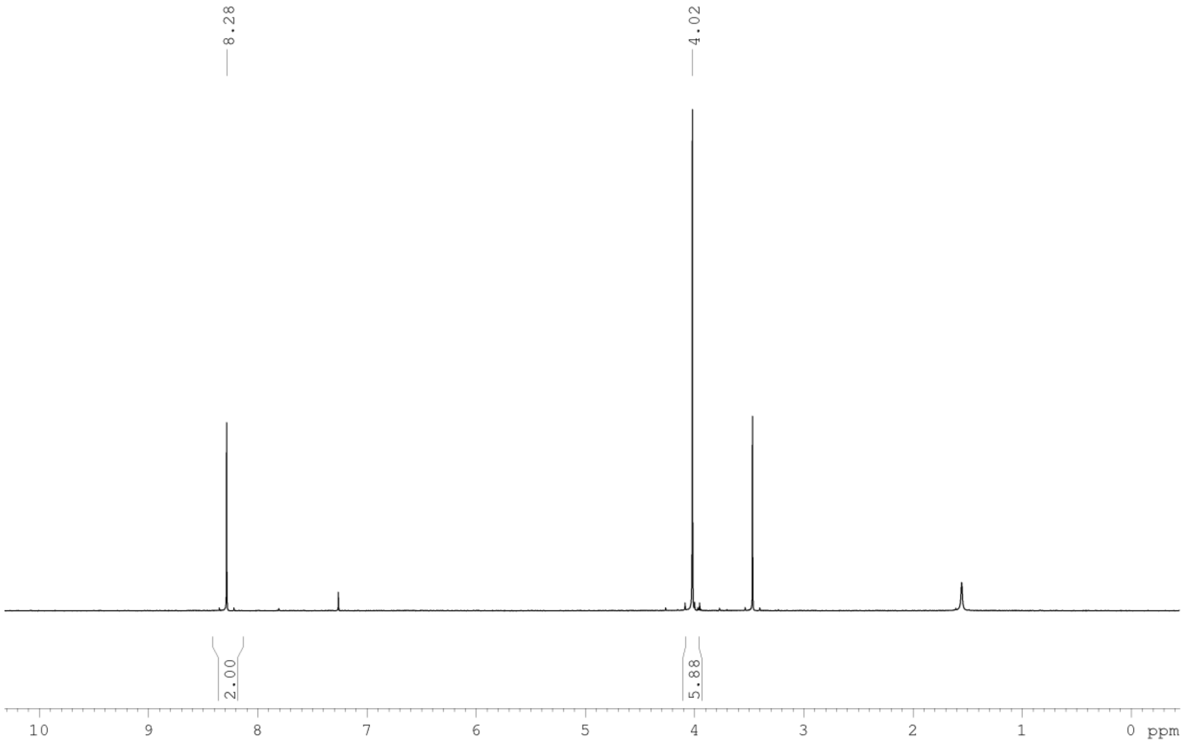


**Figure S 1. $^{1}$H-NMR spectrum of compound 1.**

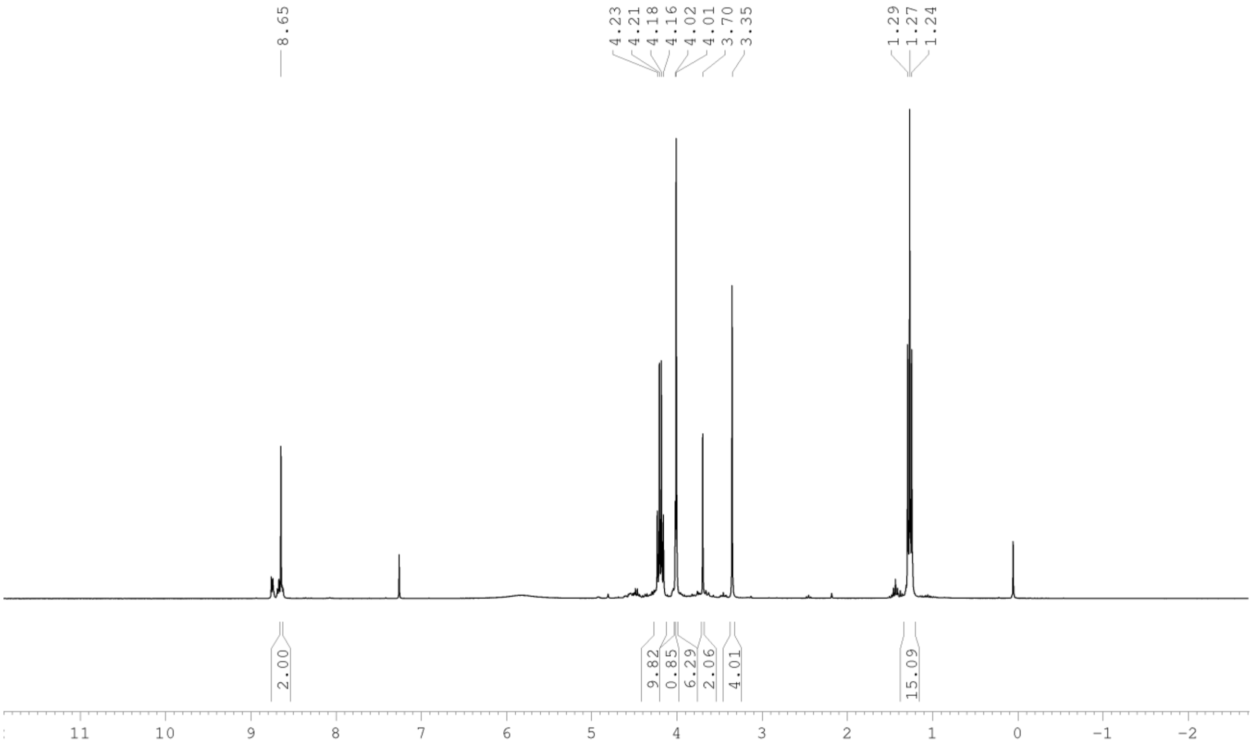


**Figure S 2. $^{1}$H-NMR spectrum of compound 2.**

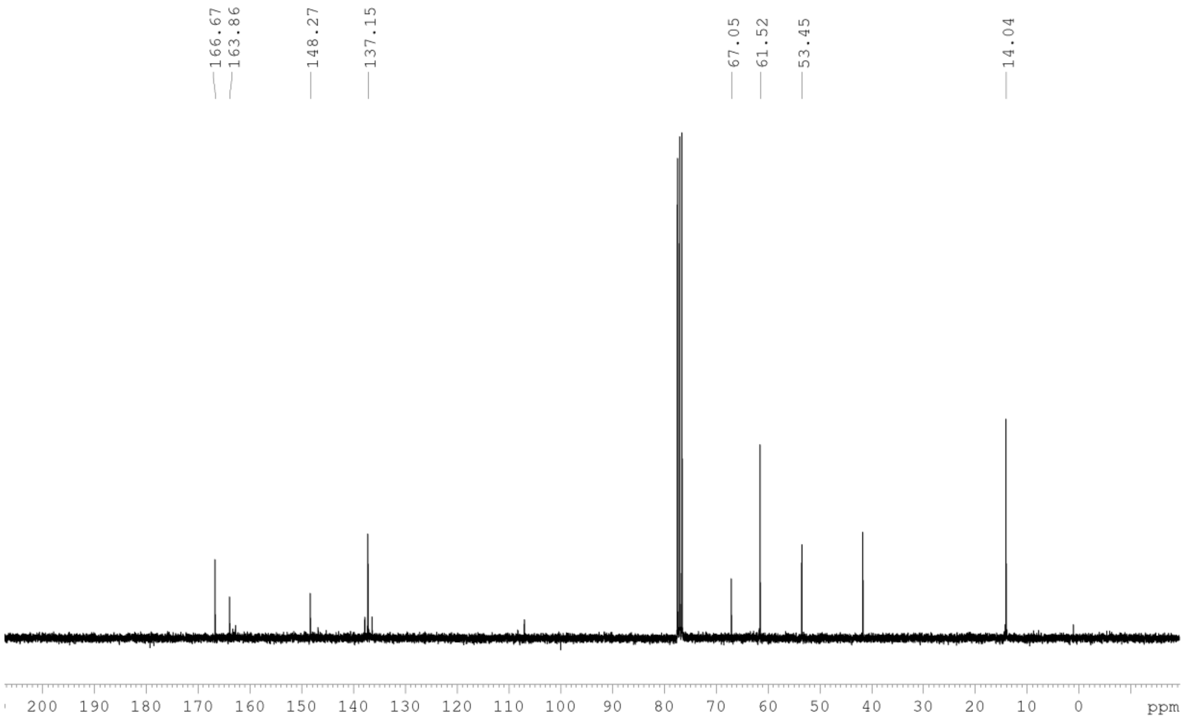


**Figure S 3. $^{13}$C-NMR spectrum of compound 2.**

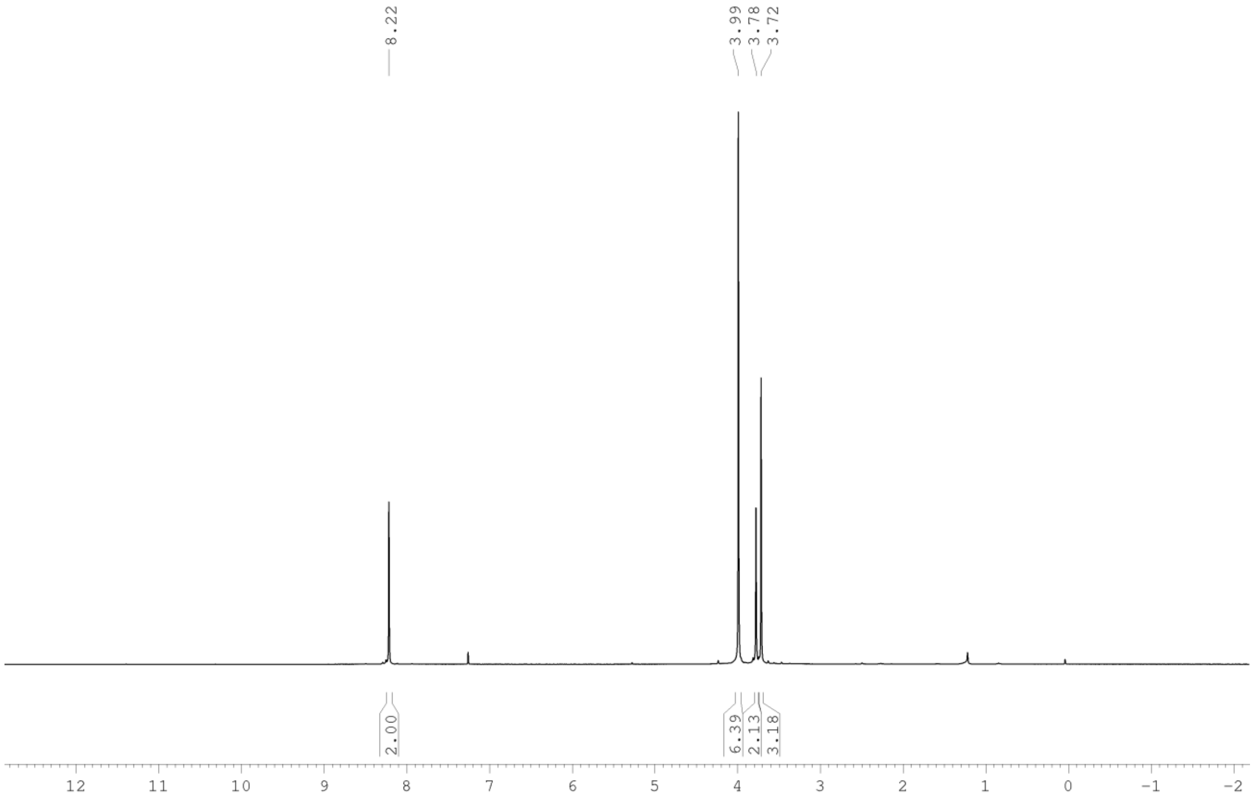


**Figure S 4. $^{1}$H-NMR spectrum of compound 3.**

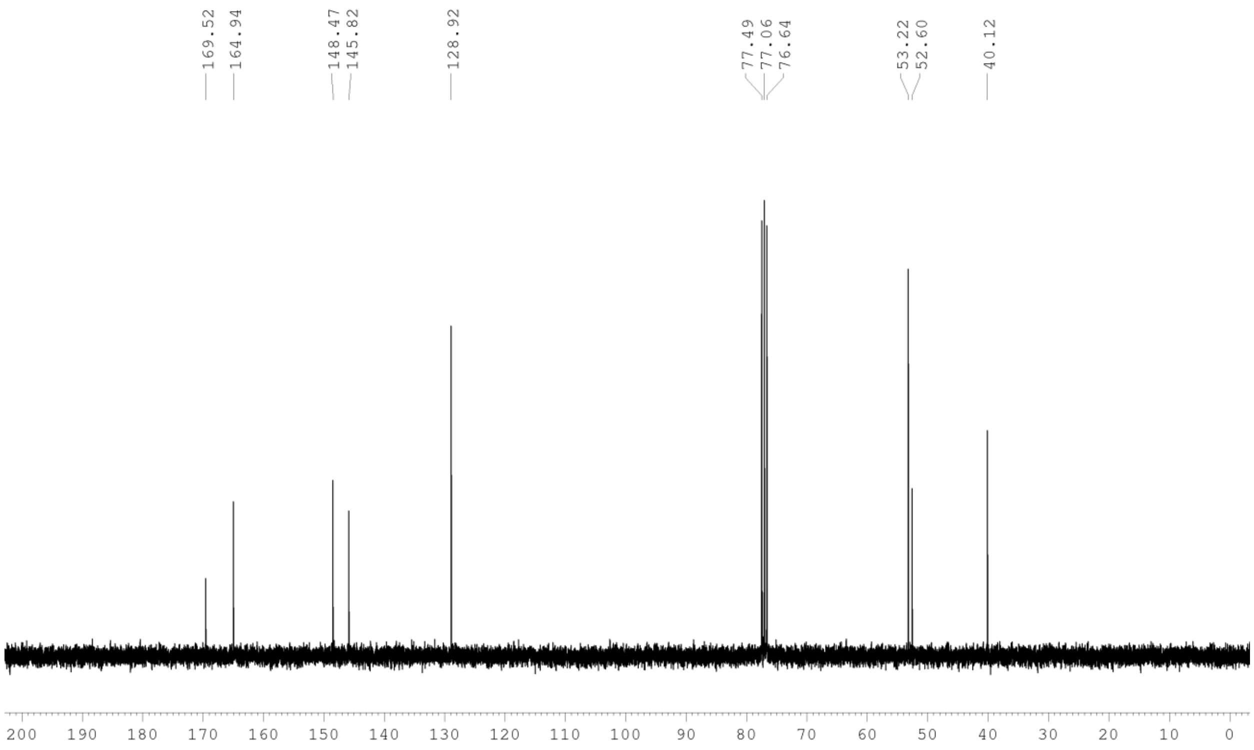


**Figure S 5. $^{13}$C-NMR spectrum of compound 3.**

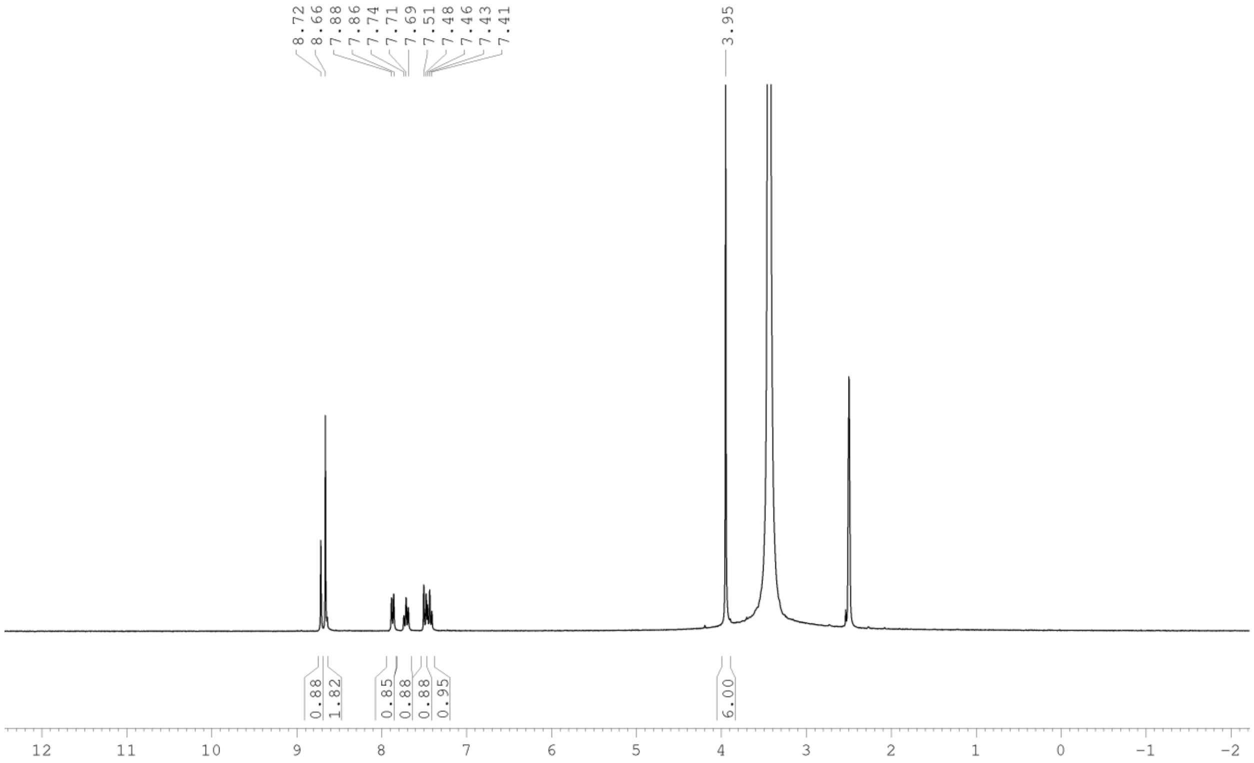


**Figure S 6. $^{1}$H-NMR spectrum of compound 4.**

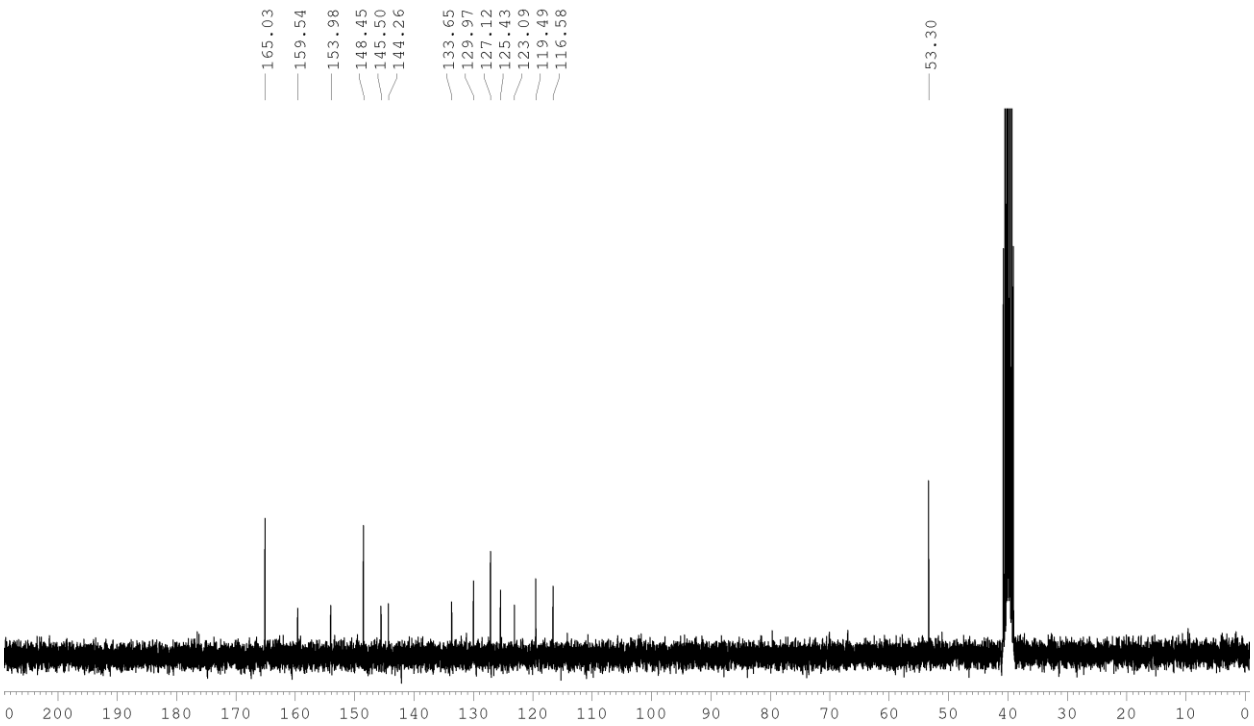


**Figure S 7. $^{13}$C-NMR spectrum of compound 4.**

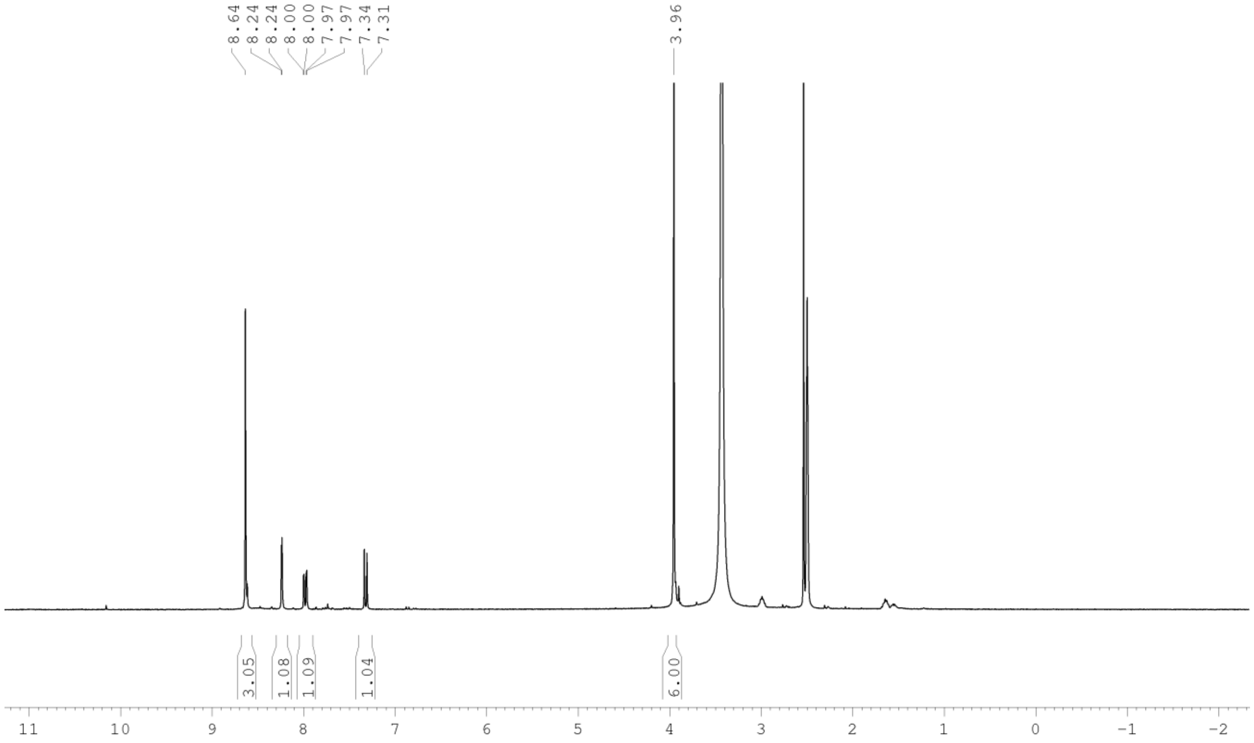


**Figure S 8. $^{1}$H-NMR spectrum of compound 5.**

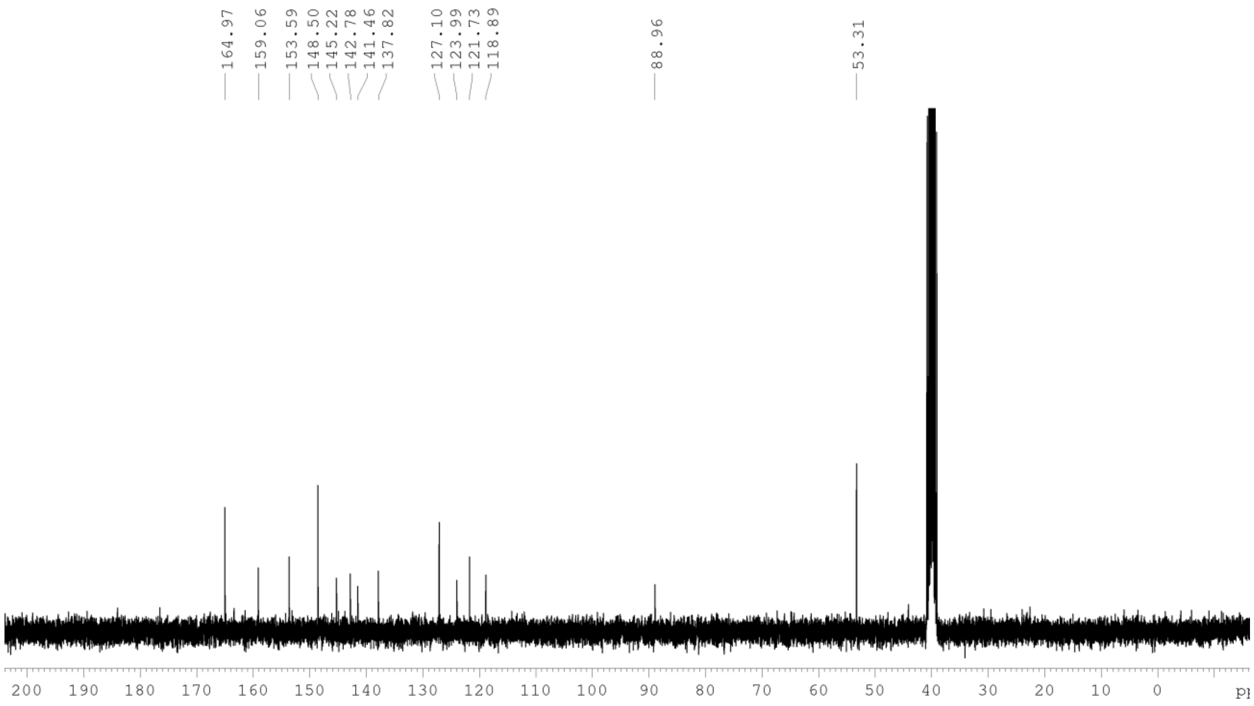


**Figure S 9. $^{13}$C-NMR spectrum of compound 5.**

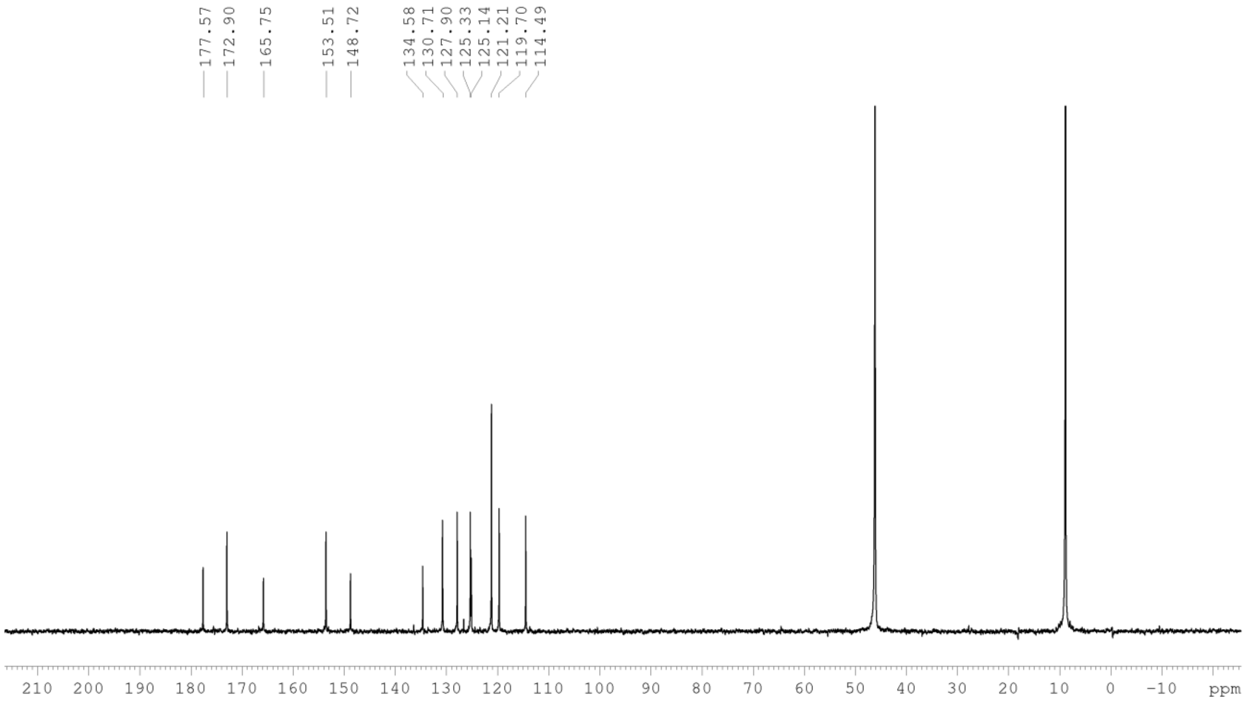


**Figure S 10. $^{13}$C-NMR spectrum of the triethylammonium salt of HL$_1$.**

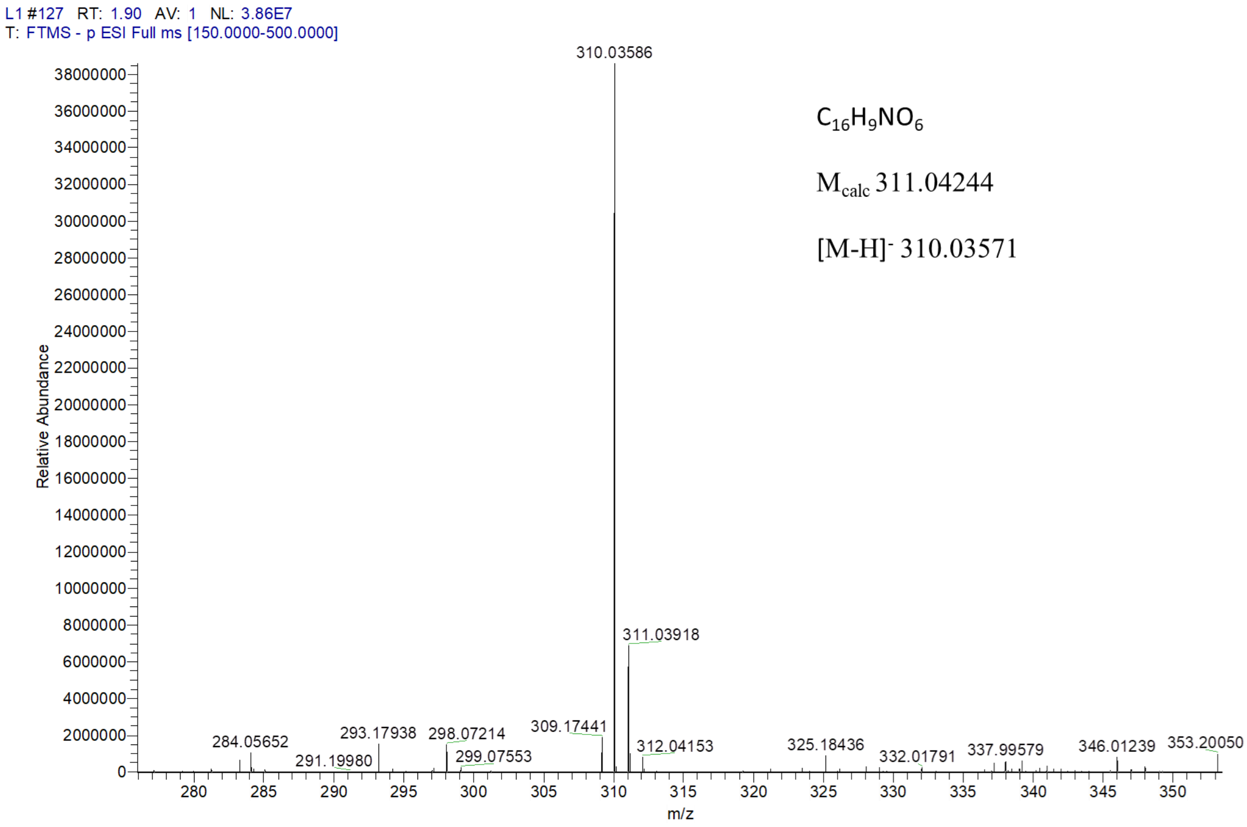


**Figure S 11. HMRS spectrum of compound $HL_1$.**

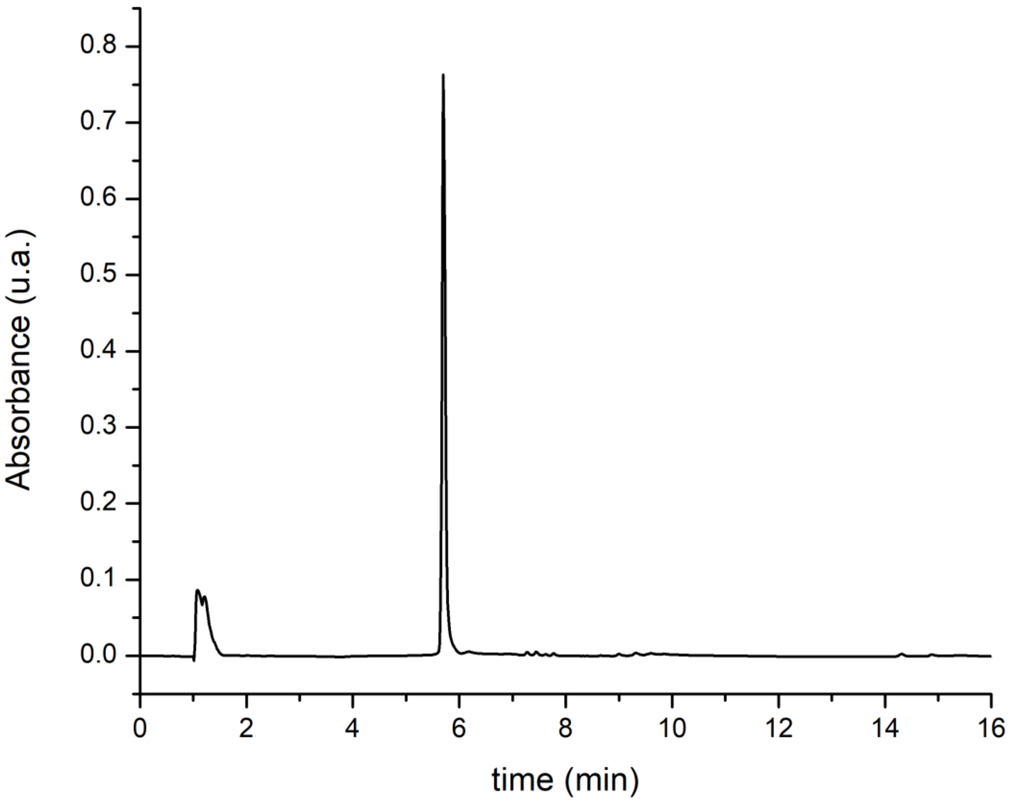


**Figure S 12. HPLC chromatogram of the triethylammonium salt of $HL_1$.**

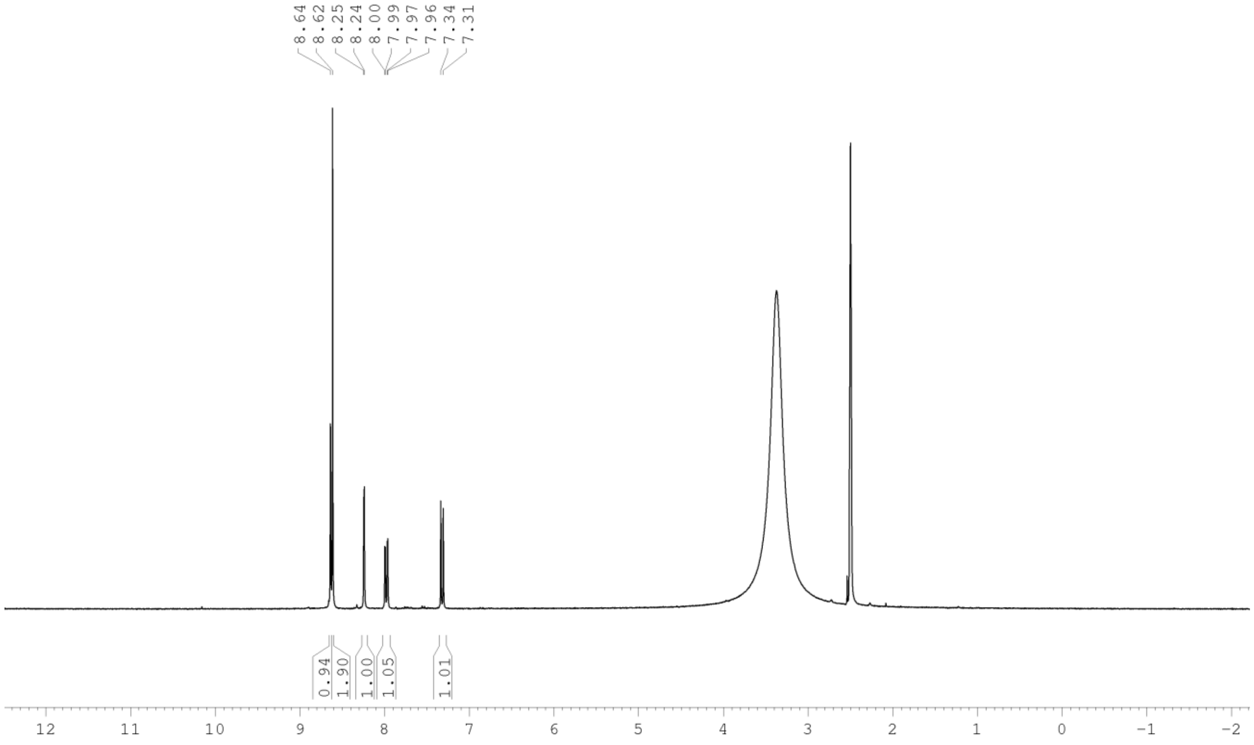


**Figure S 13. $^1$H-NMR spectrum of compound HL$_2$.**

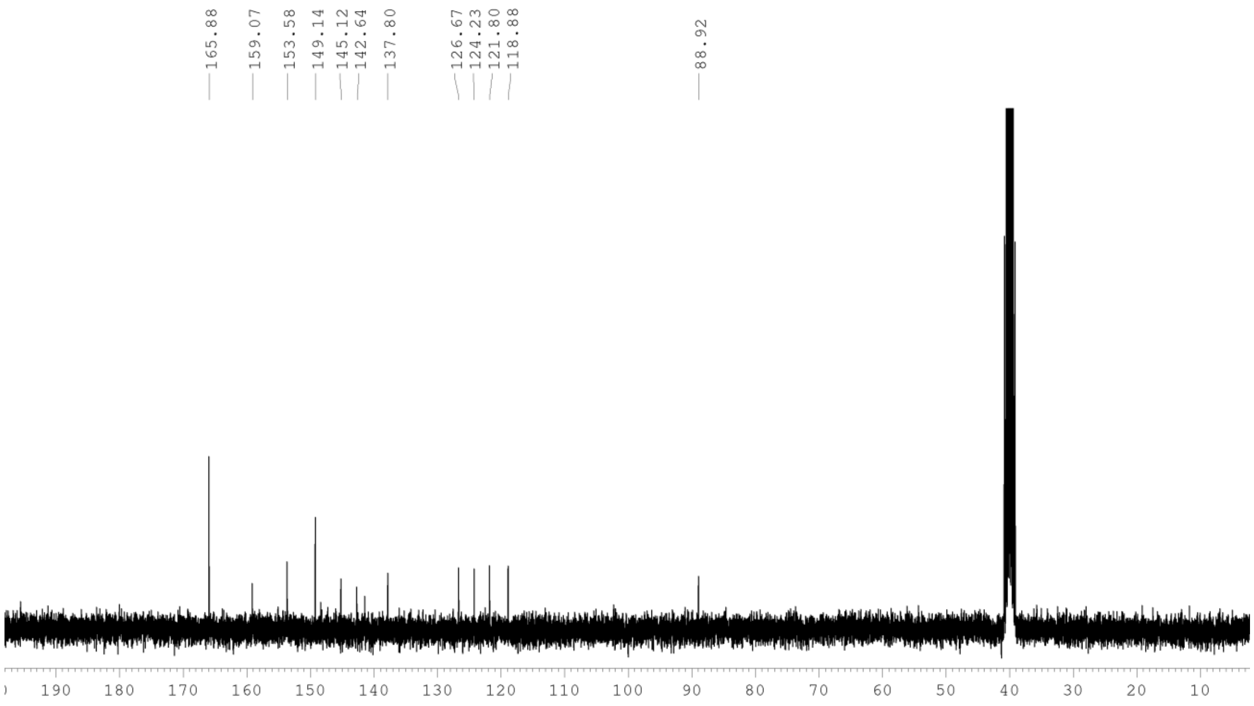


**Figure S 14. $^{13}$C-NMR spectrum of compound HL$_2$.**

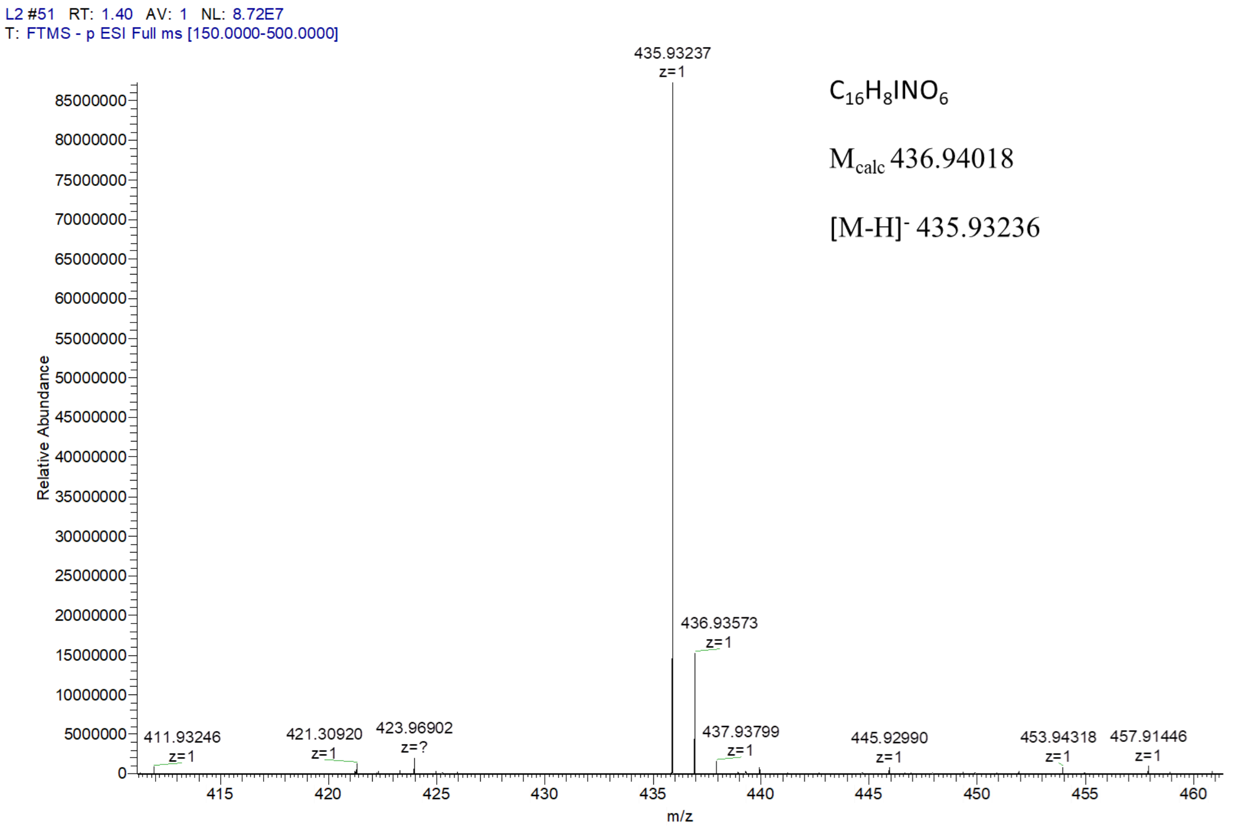


**Figure S 15. HMRS spectrum of compound $HL_2$.**

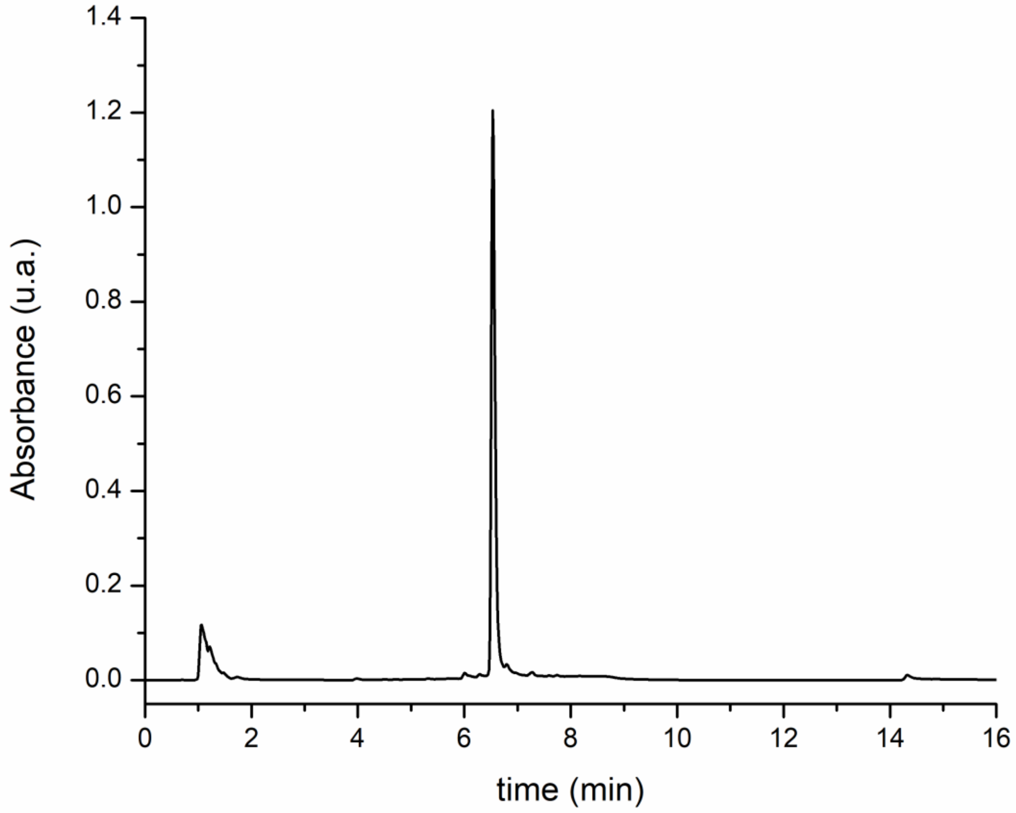


**Figure S 16. HPLC chromatogram of the triethylammonium salt of $HL_2$.**

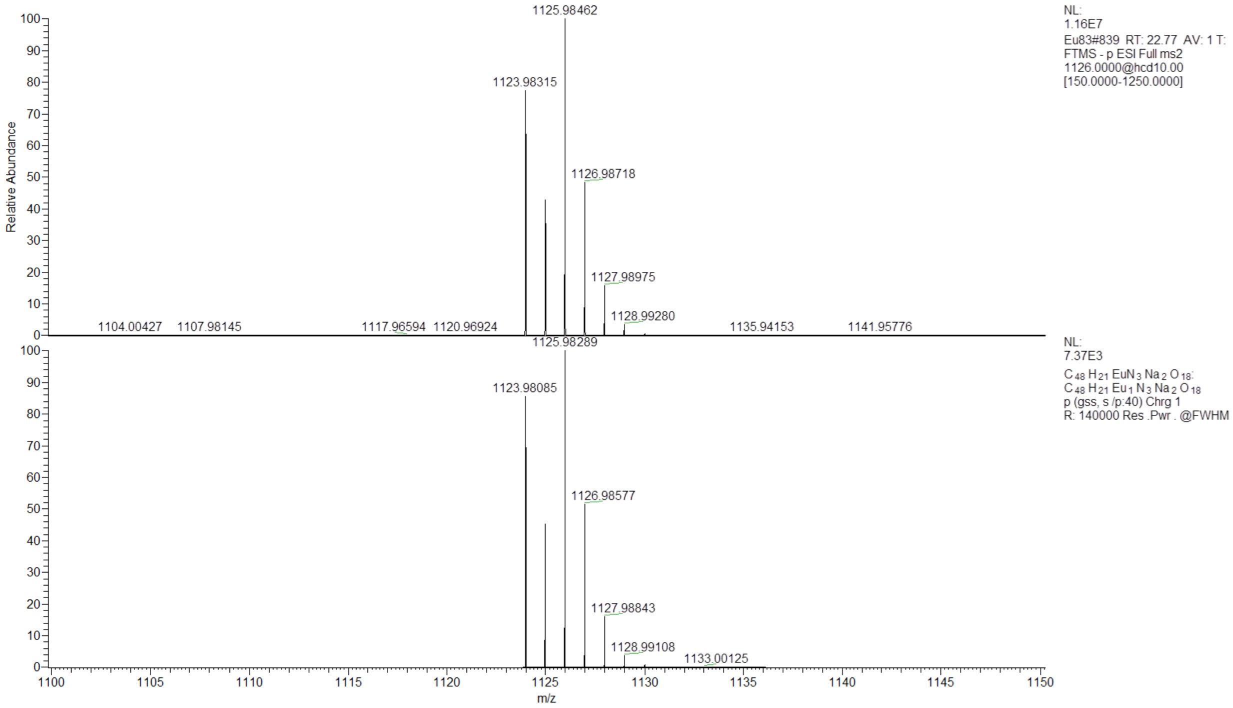


**Figure S 17. HRMS spectrum of $Na_3[Eu(L_1)_3]$ complex (above) with the relative calculated one (below).**

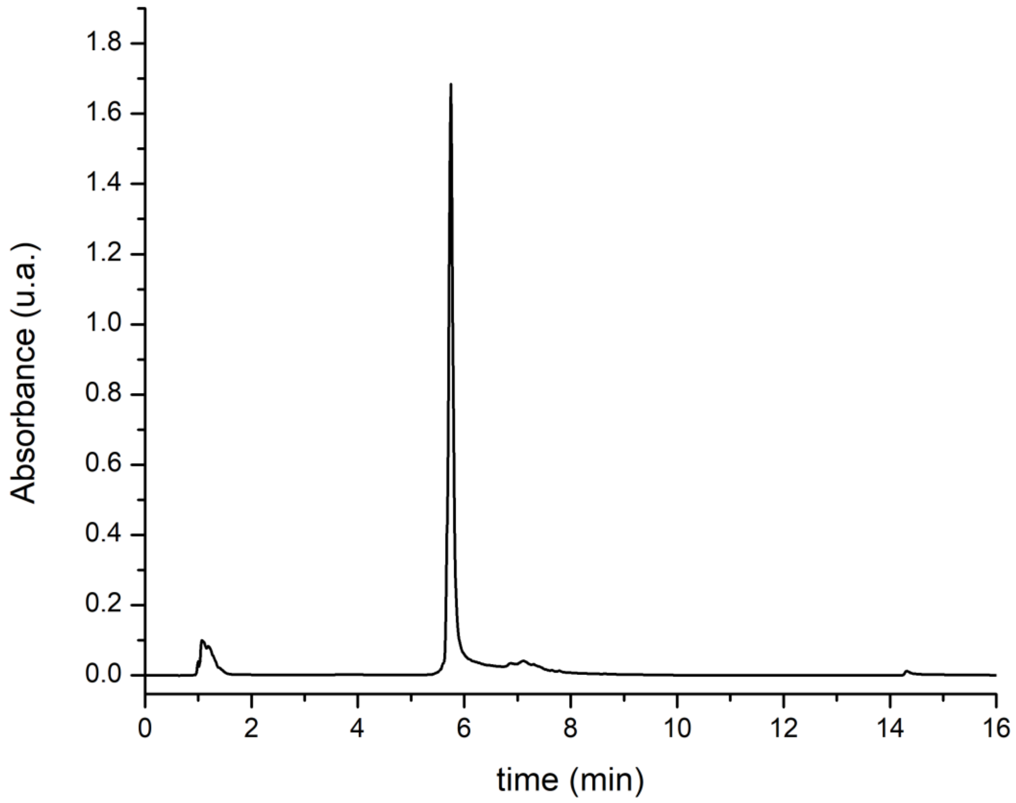


**Figure S 18. HPLC chromatogram of $Na_3[Eu(L_1)_3]$ complex.**

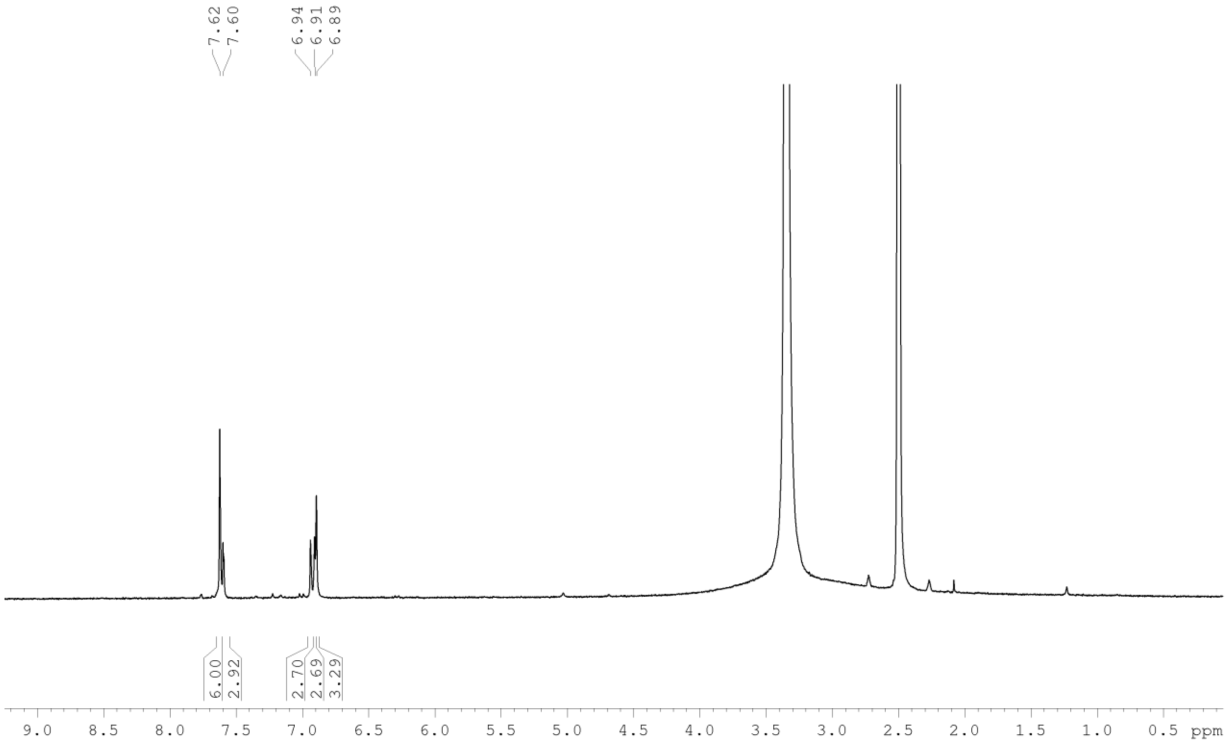


**Figure S 19. $^{1}H$-NMR spectrum of $Na_3[Eu(L2)_3]$ complex.**

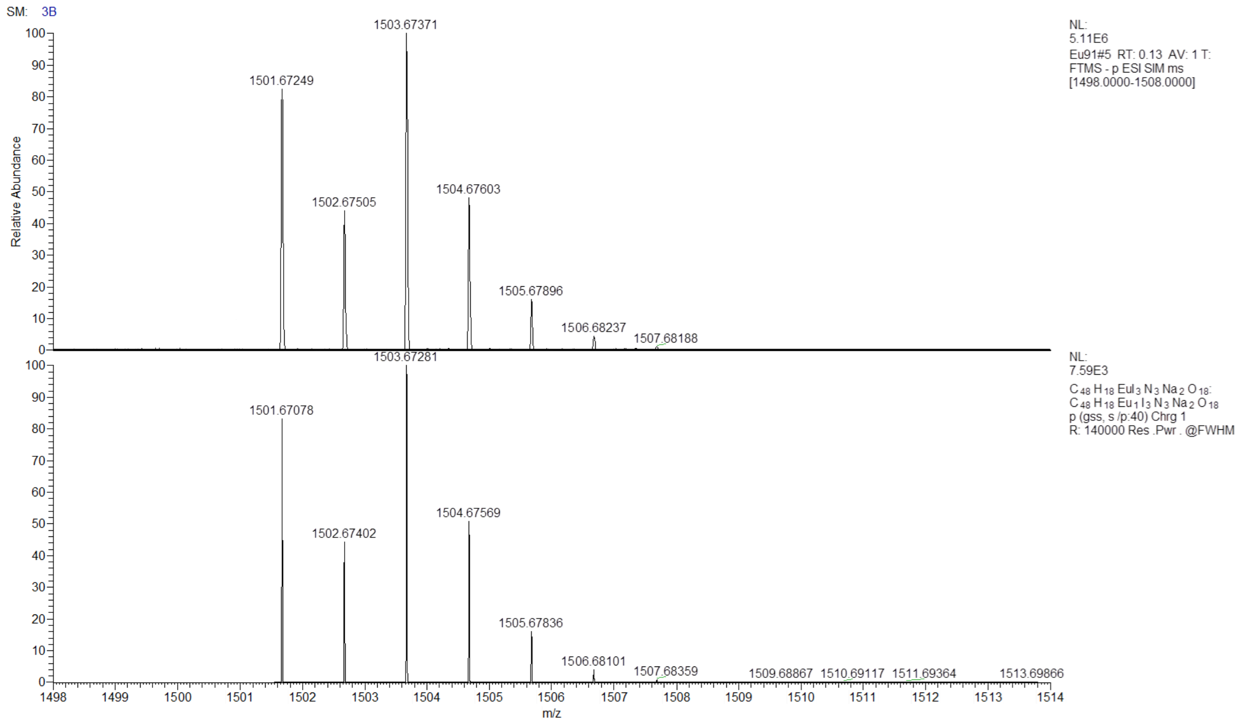


**Figure S 20. HRMS spectrum of $Na_3[Eu(L_2)_3]$ complex (above) with the relative calculated one (below).**

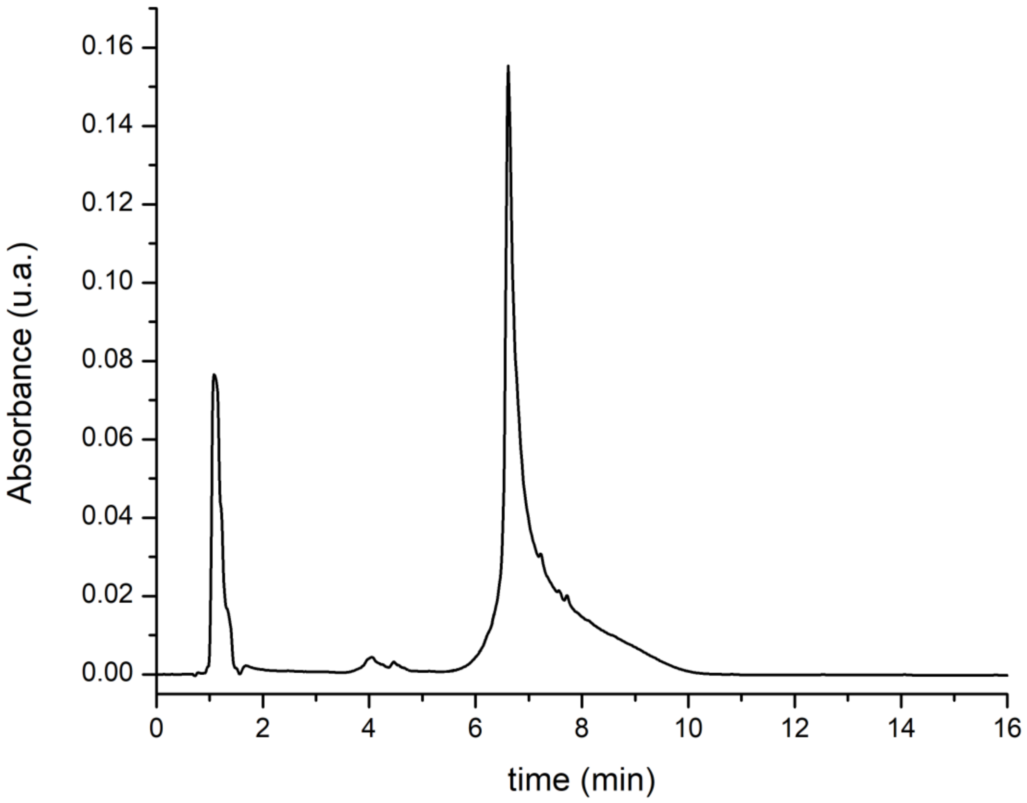


**Figure S 21. HPLC chromatogram of $Na_3[Eu(L_2)_3]$ complex.**

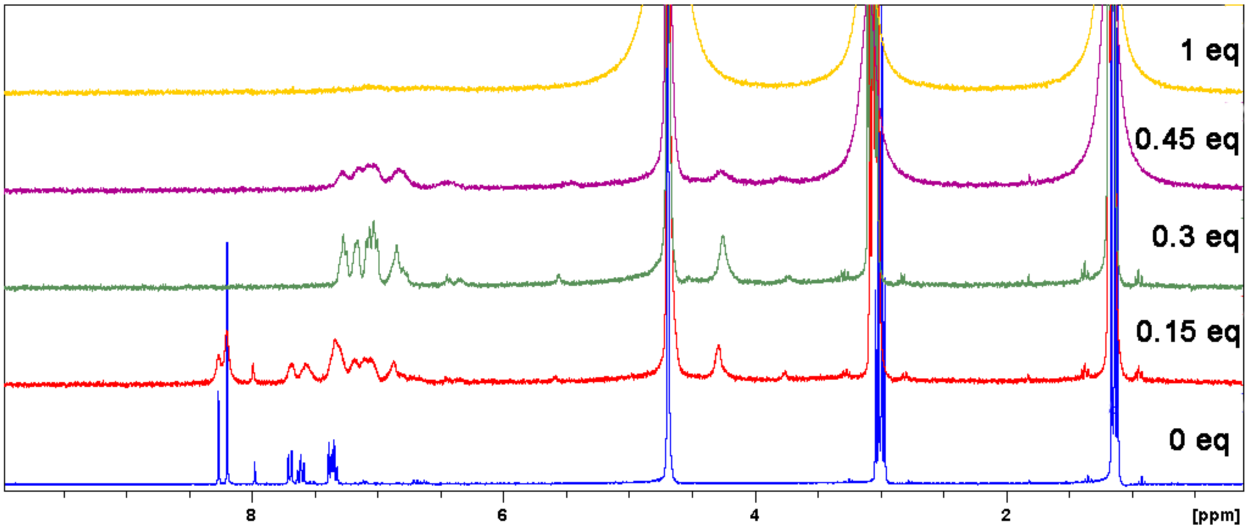


**Figure S 22.** **$^{1}H$-NMR titration (400 MHz, $D_2O$) of $L_1$ (triethylammonium salt) with $Eu^{3+}$ (chloride hexahydrate salt).**

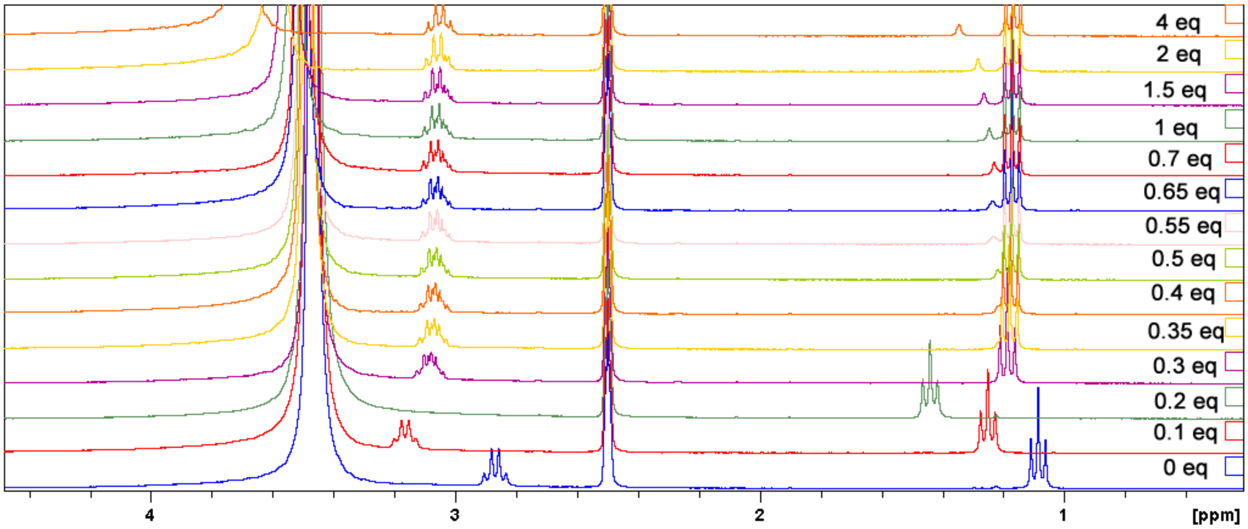


**Figure S 23.** **$^{1}H$-NMR titration (400 MHz, DMSOd) of $L_1$ (triethylammonium salt) with $Eu^{3+}$ (chloride hexahydrate salt), particular of the counterion dynamic.**

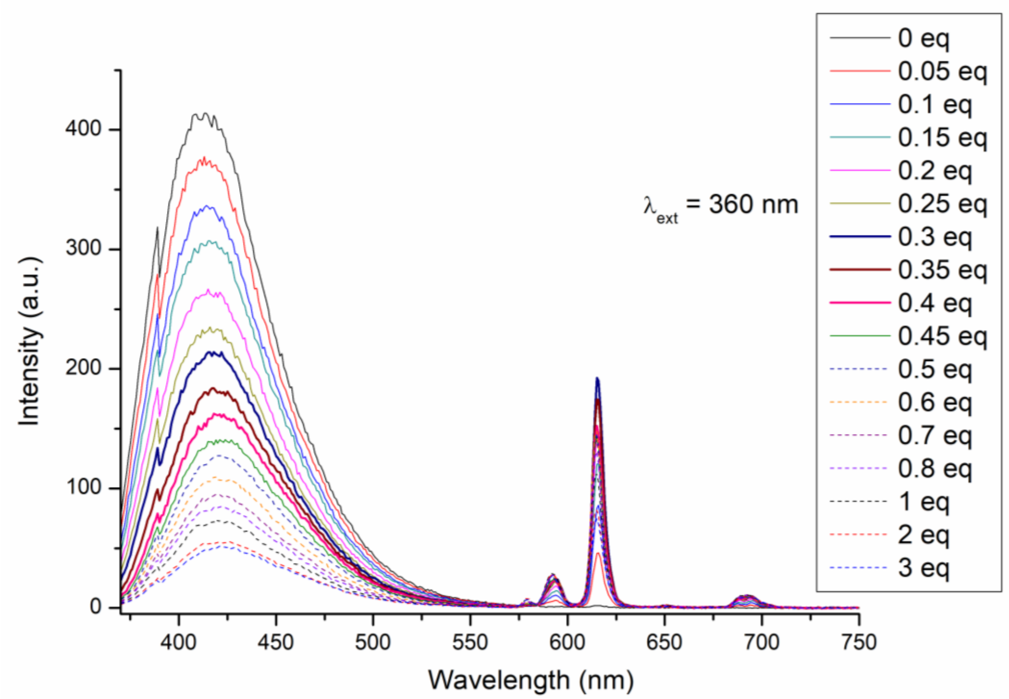


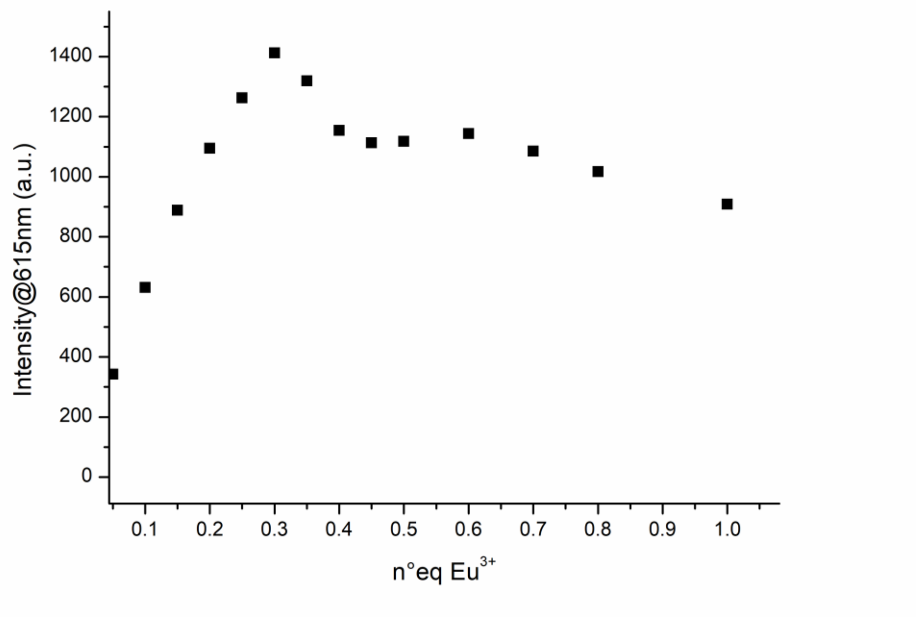


**Figure S 24. Fluorescence titration spectra of $L_1^{2-}$ in Tris HCl with $Eu^{3+}$ (above) and titration curve monitoring the fluorescence at 615 nm (below).**

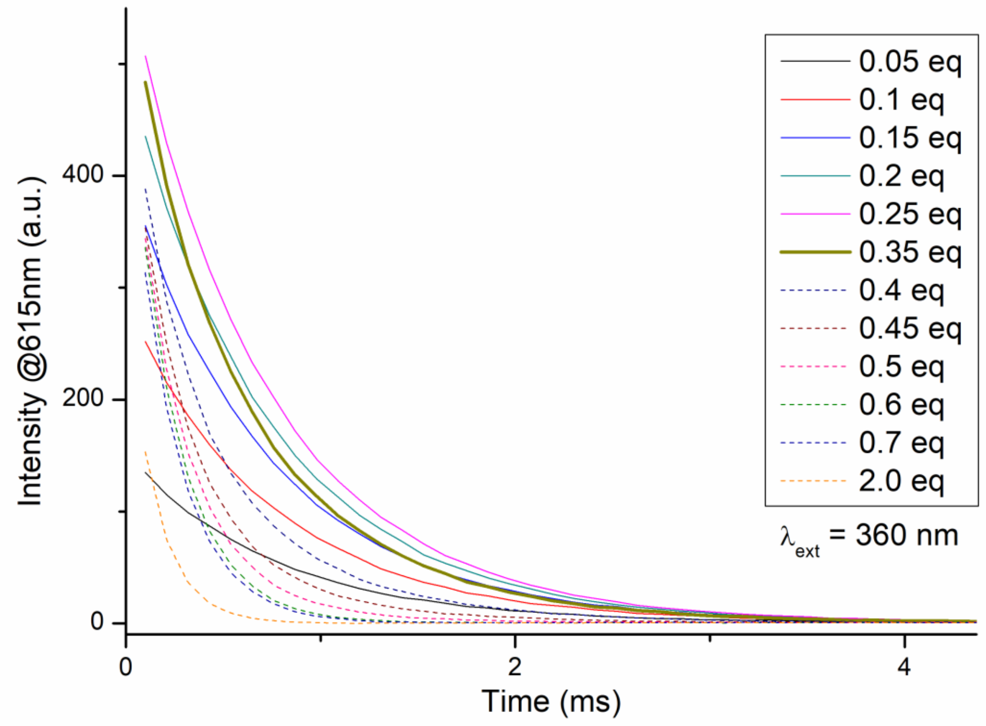

0.05 eq
0.1 eq
0.15 eq
0.2 eq
0.25 eq
0.35 eq
0.4 eq
0.45 eq
0.5 eq
0.6 eq
0.7 eq
2.0 eq
λext = 360 nm
Intensity @615nm (a.u.)
400
200
0
0
2
4
Time (ms)

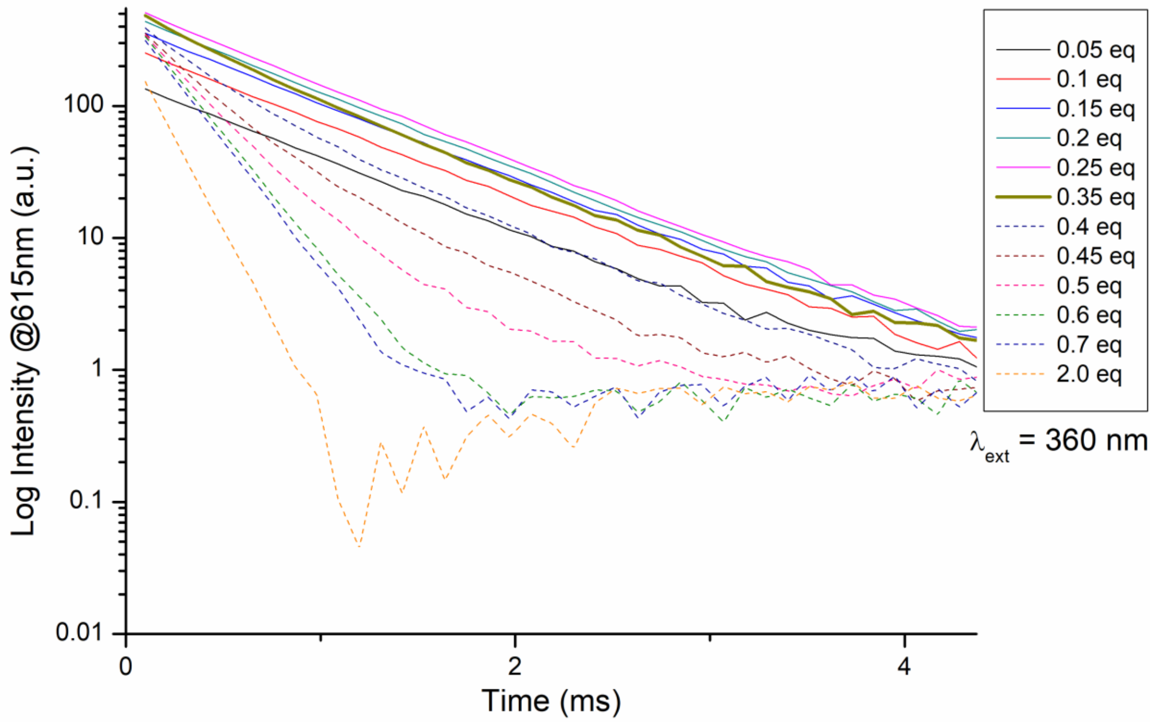


**Figure S 25. Fluorescence decay fitting of $L_1^{2-}$ in Tris HCl with increasing amounts of $Eu^{3+}$ monitoring at 615 nm and exciting at 360 nm.**

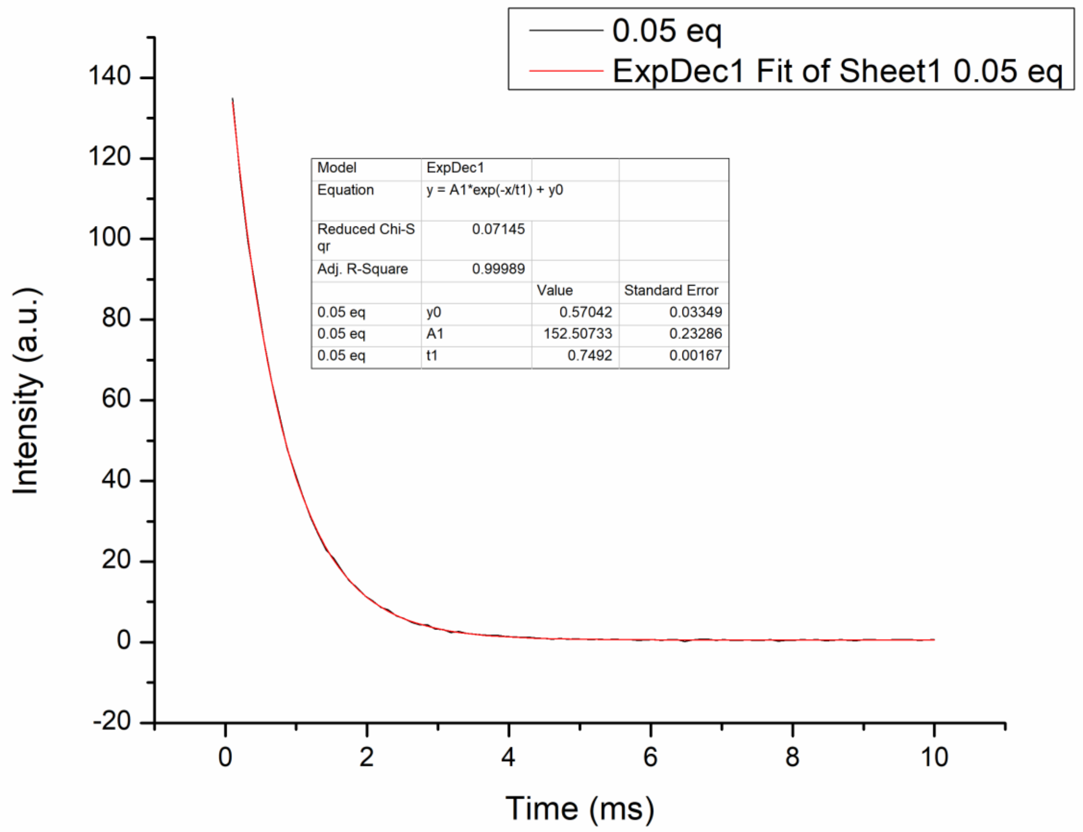
0.05 eq
ExpDec1 Fit of Sheet1 0.05 eq
Model
ExpDec1
Equation
y = A1*exp(-x/t1) + y0
Reduced Chi-Sqr
0.07145
Adj. R-Square
0.99989
Value
Standard Error
0.05 eq
y0
0.57042
0.03349
0.05 eq
A1
152.50733
0.23286
0.05 eq
t1
0.7492
0.00167
Intensity (a.u.)
Time (ms)
140
120
100
80
60
40
20
0
-20
0
2
4
6
8
10

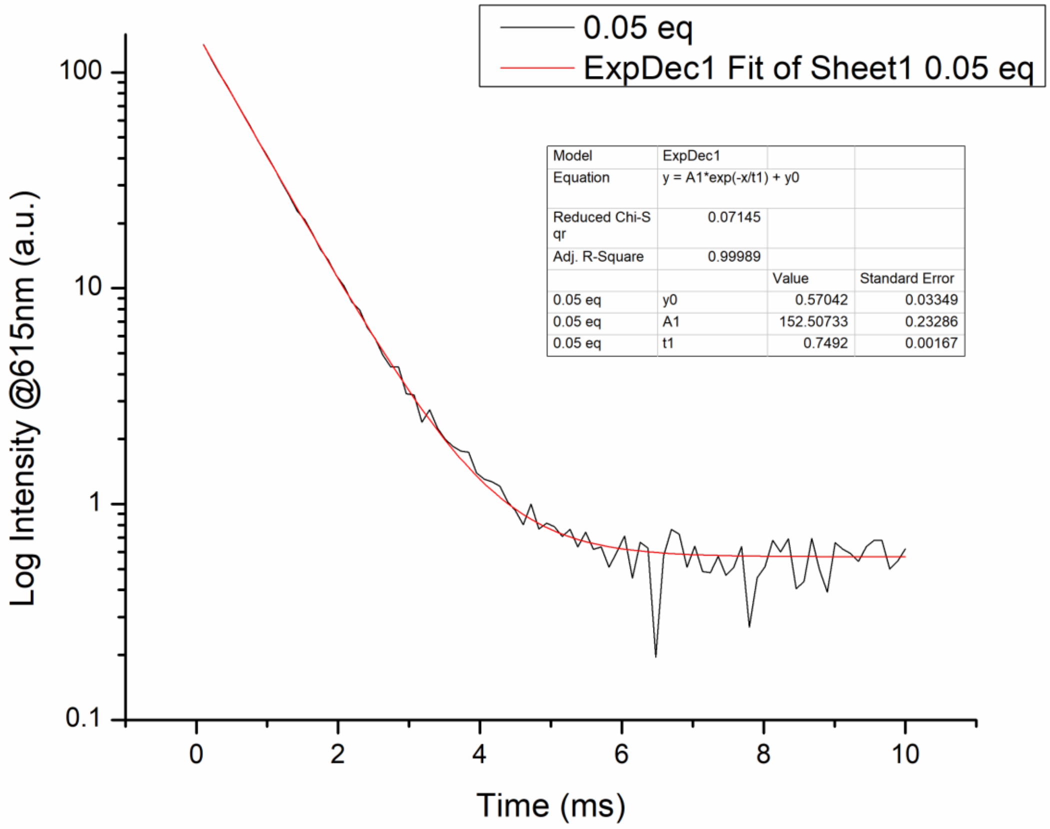


**Figure S 26. Fluorescence decay fitting of $L_1^{2-}$ in Tris HCl with increasing amounts of $Eu^{3+}$ monitoring at 615 nm and exciting at 360 nm: 0.05 eq**

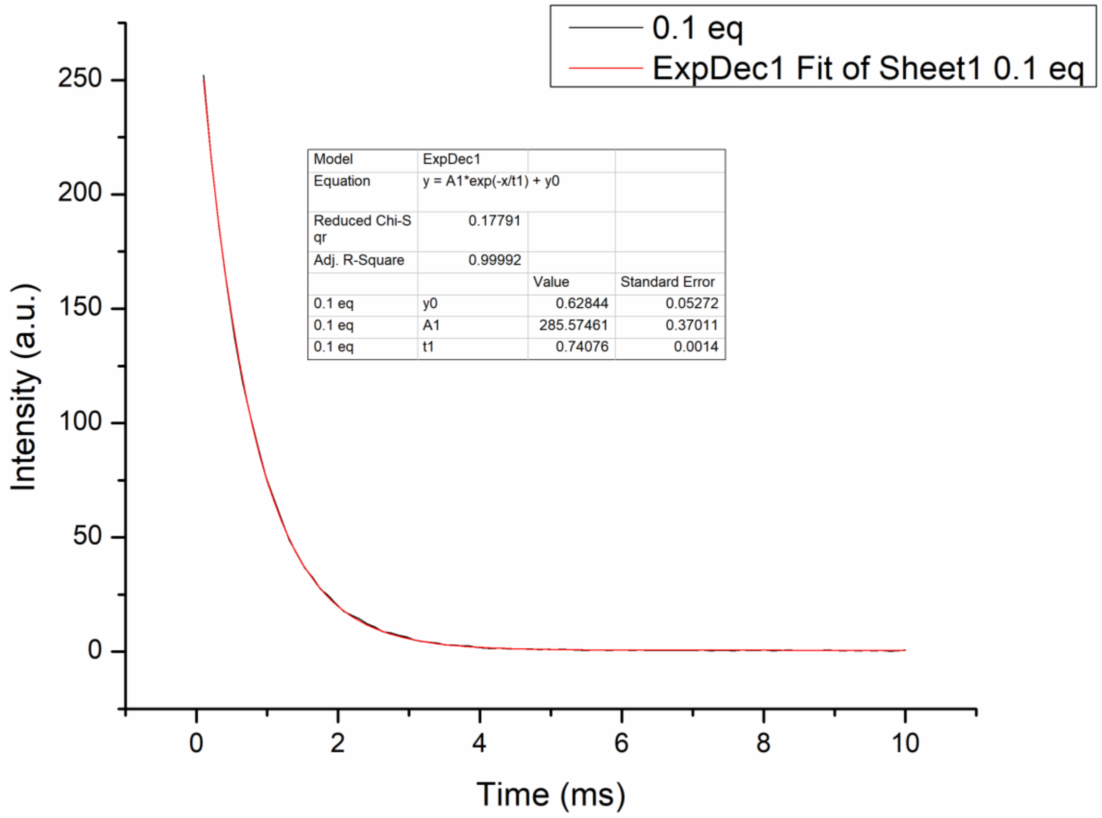
0.1 eq
ExpDec1 Fit of Sheet1 0.1 eq
Model ExpDec1
Equation y = A1*exp(-x/t1) + y0
Reduced Chi-Sqr 0.17791
Adj. R-Square 0.99992
Value Standard Error
0.1 eq y0 0.62844 0.05272
0.1 eq A1 285.57461 0.37011
0.1 eq t1 0.74076 0.0014
Intensity (a.u.)
Time (ms)
0
50
100
150
200
250
0
2
4
6
8
10

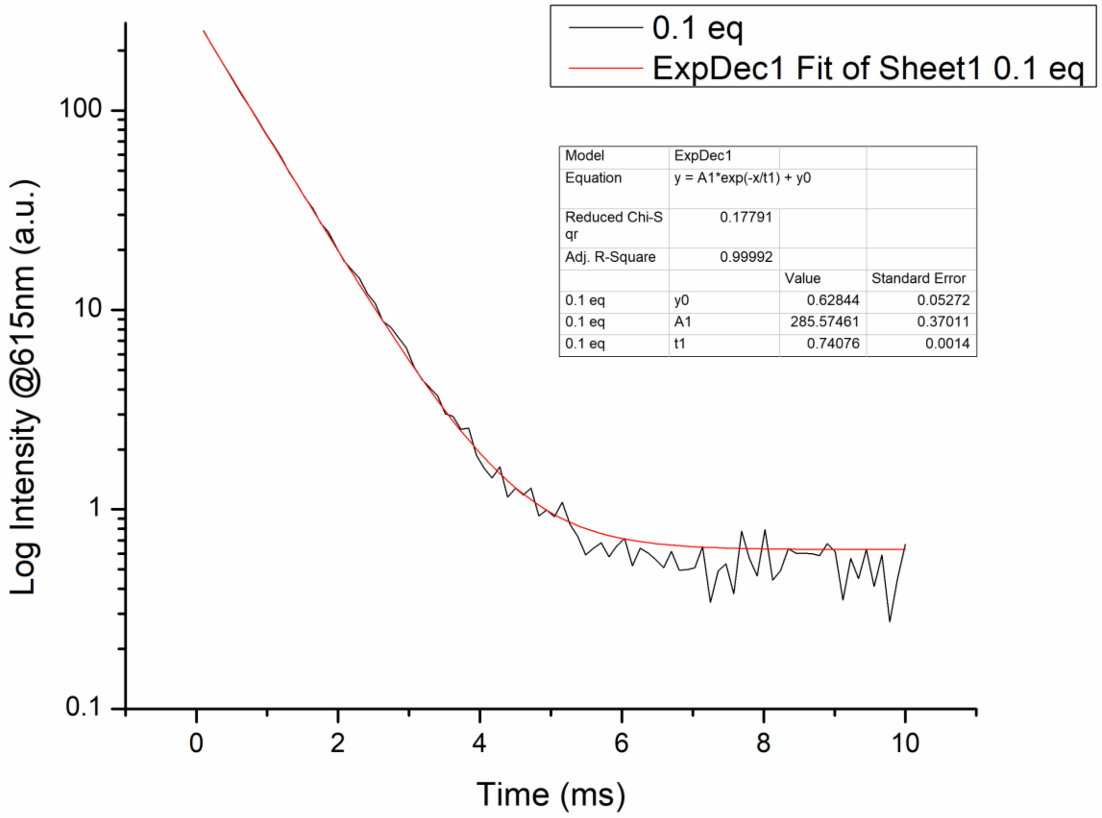


**Figure S 27. Fluorescence decay fitting of $L_1^{2-}$ in Tris HCl with increasing amounts of $Eu^{3+}$ monitoring at 615 nm and exciting at 360 nm: 0.1 eq**

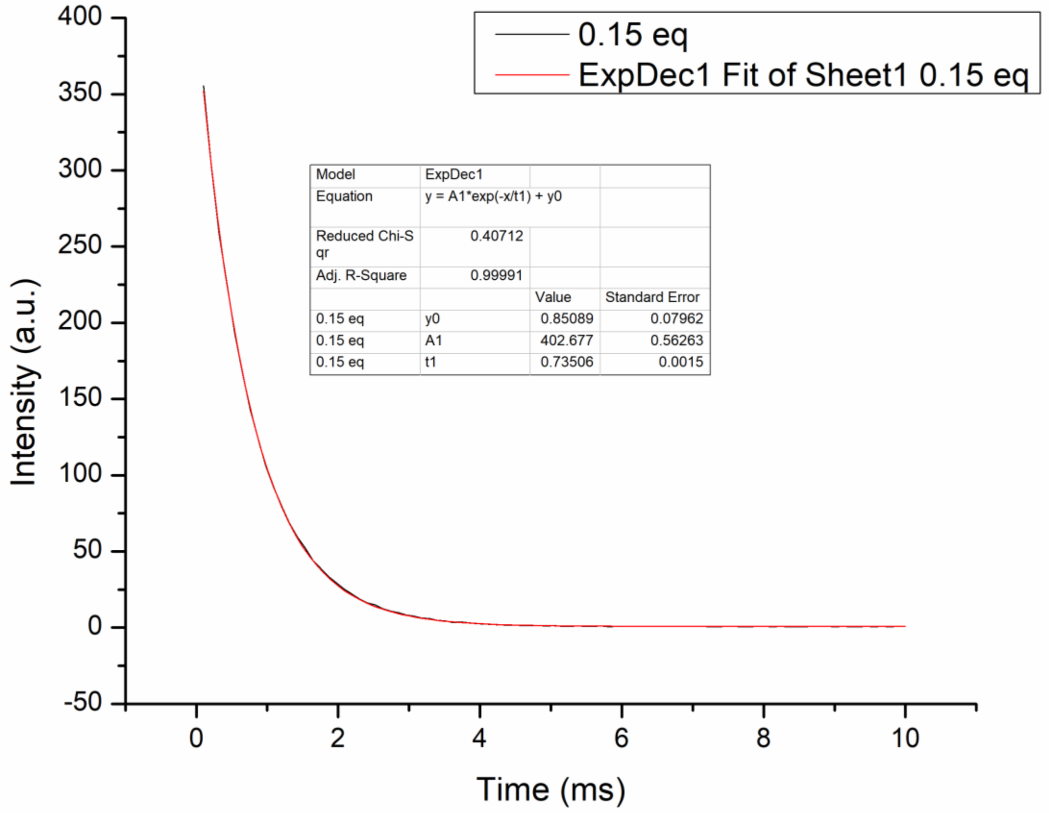

0.15 eq
ExpDec1 Fit of Sheet1 0.15 eq
Model
ExpDec1
Equation
y = A1*exp(-x/t1) + y0
Reduced Chi-Sqr
0.40712
Adj. R-Square
0.99991
Value
Standard Error
0.15 eq
y0
0.85089
0.07962
0.15 eq
A1
402.677
0.56263
0.15 eq
t1
0.73506
0.0015
Intensity (a.u.)
Time (ms)
400
350
300
250
200
150
100
50
0
-50
0
2
4
6
8
10

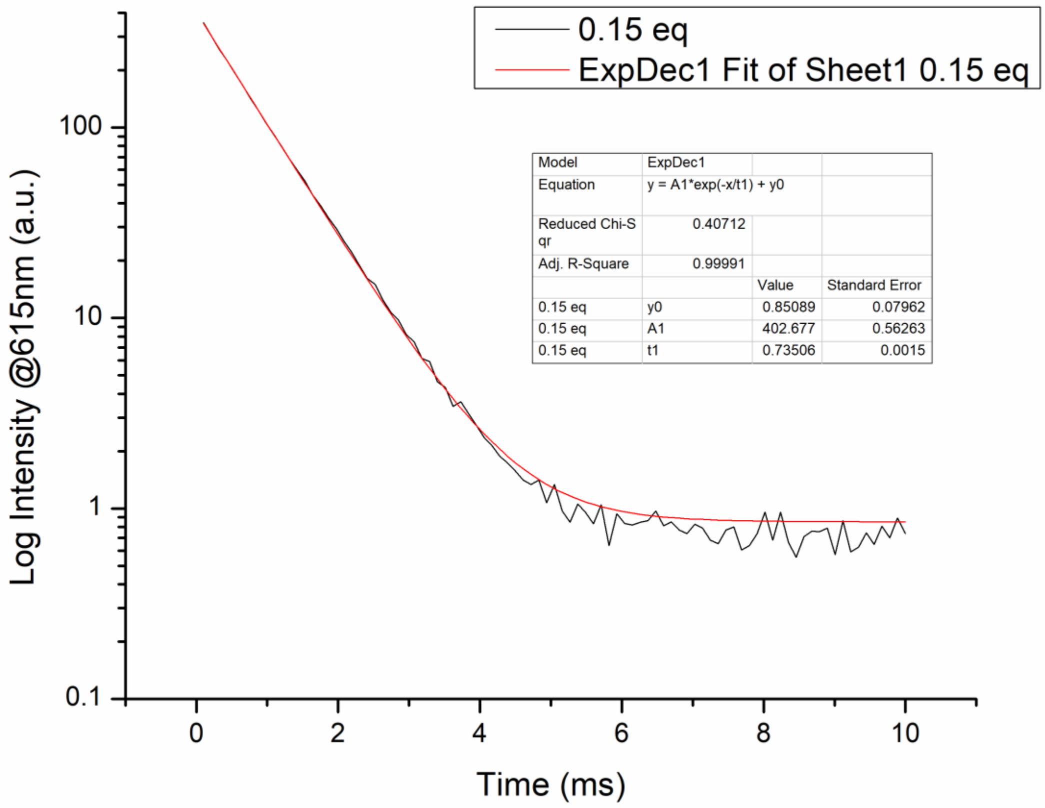


**Figure S 28. Fluorescence decay fitting of $L_1^{2-}$ in Tris HCl with increasing amounts of $Eu^{3+}$ monitoring at 615 nm and exciting at 360 nm: 0.15 eq**

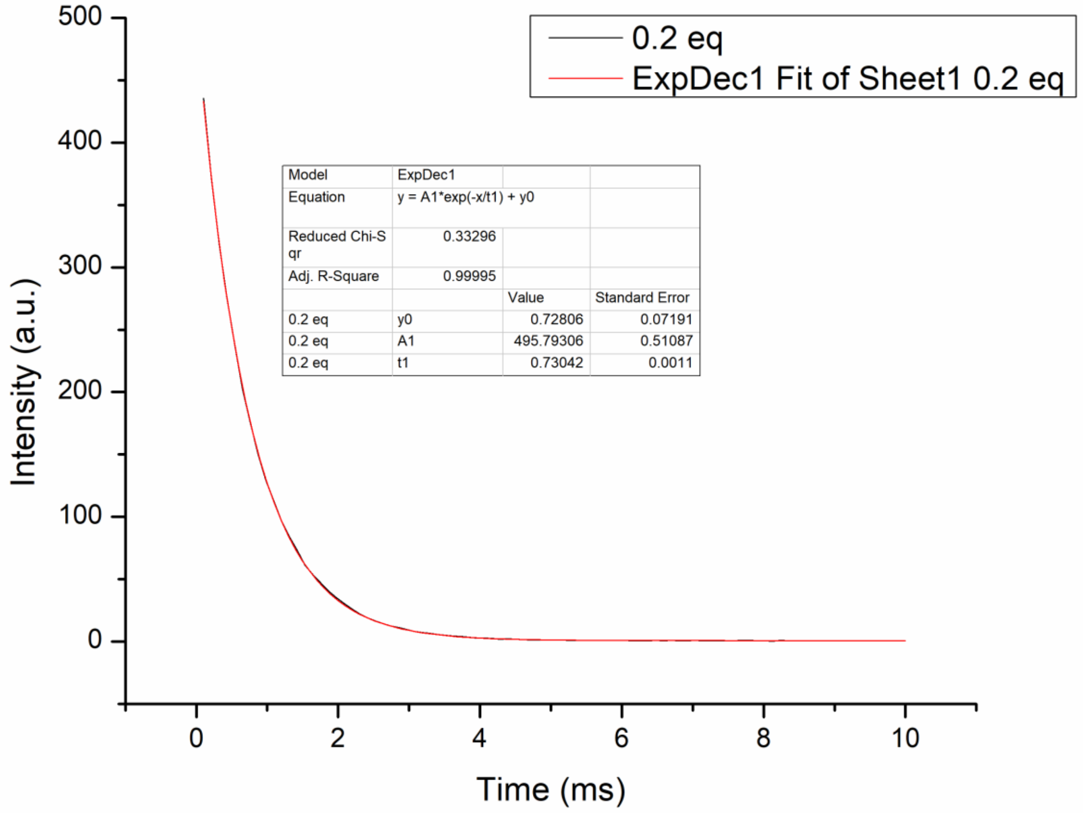
0.2 eq
ExpDec1 Fit of Sheet1 0.2 eq
Model
ExpDec1
Equation
y = A1*exp(-x/t1) + y0
Reduced Chi-Sqr
0.33296
Adj. R-Square
0.99995
Value
Standard Error
0.2 eq
y0
0.72806
0.07191
0.2 eq
A1
495.79306
0.51087
0.2 eq
t1
0.73042
0.0011
Intensity (a.u.)
Time (ms)
500
400
300
200
100
0
0
2
4
6
8
10

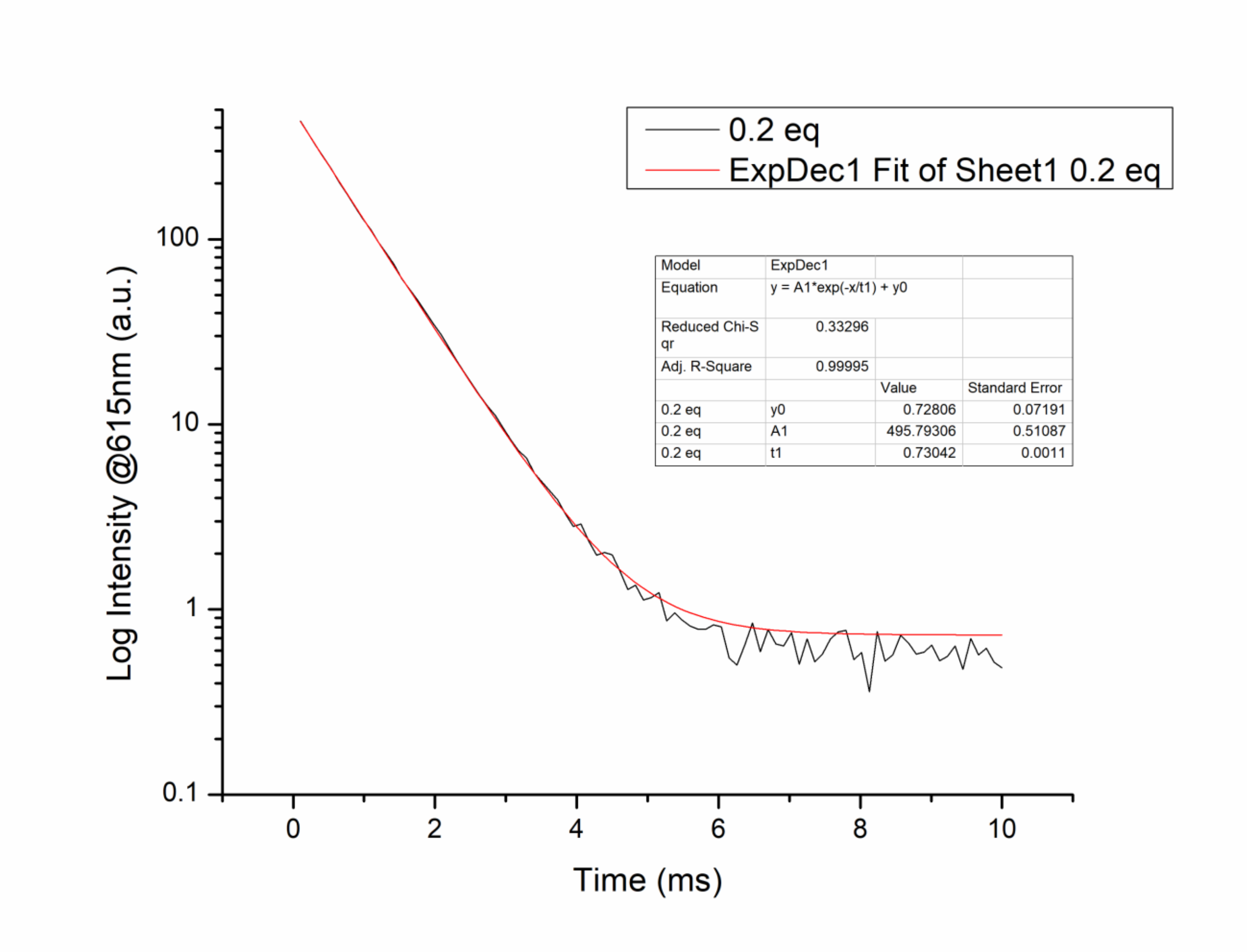


**Figure S 29. Fluorescence decay fitting of $L_1^{2-}$ in Tris HCl with increasing amounts of $Eu^{3+}$ monitoring at 615 nm and exciting at 360 nm: 0.2 eq**

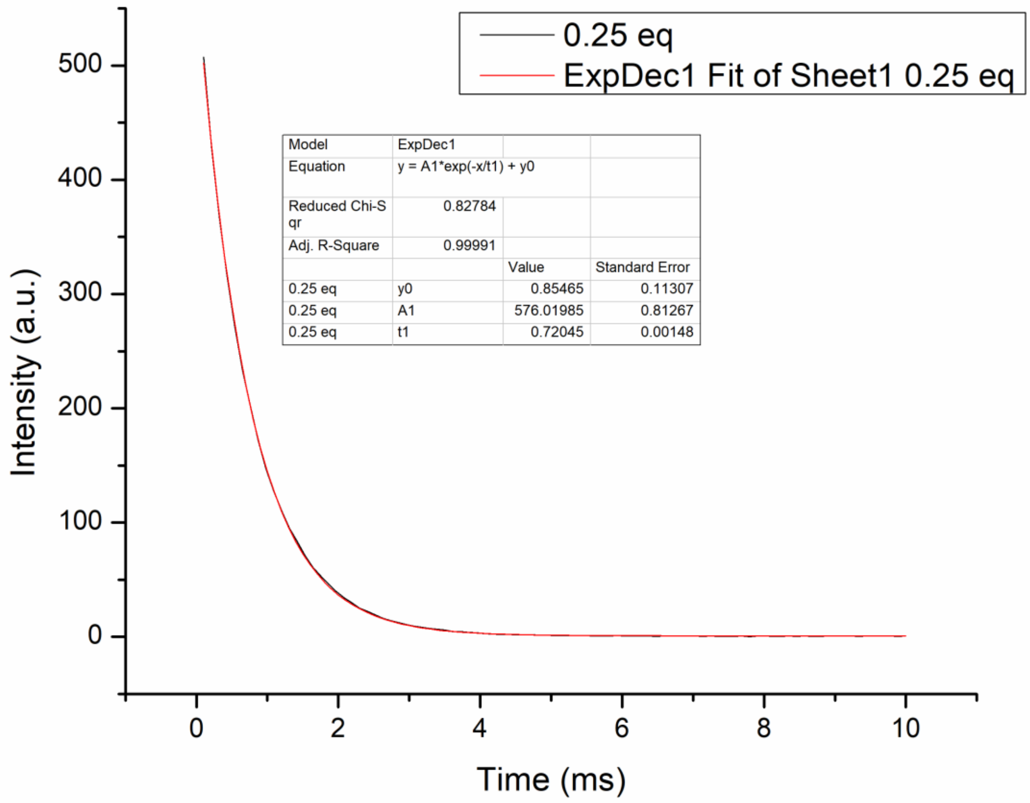

0.25 eq
ExpDec1 Fit of Sheet1 0.25 eq
Model ExpDec1
Equation y = A1*exp(-x/t1) + y0
Reduced Chi-Sqr 0.82784
Adj. R-Square 0.99991
Value Standard Error
0.25 eq y0 0.85465 0.11307
0.25 eq A1 576.01985 0.81267
0.25 eq t1 0.72045 0.00148
Intensity (a.u.)
Time (ms)

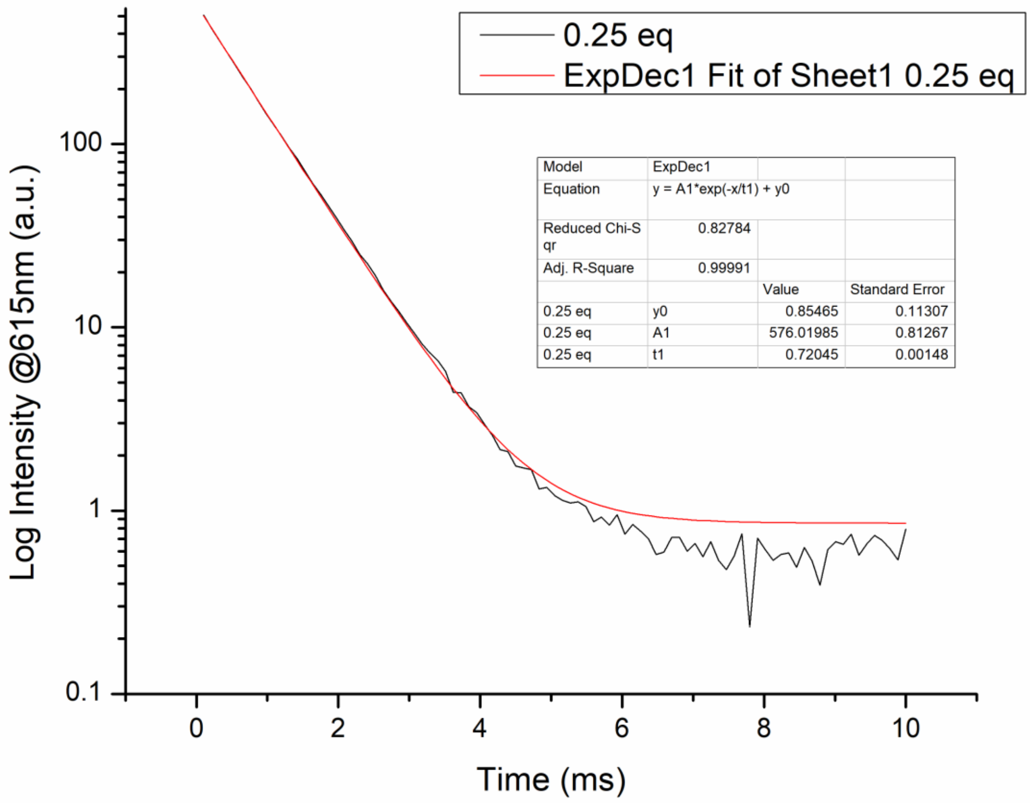


**Figure S 30. Fluorescence decay fitting of $L_1^{2-}$ in Tris HCl with increasing amounts of $Eu^{3+}$ monitoring at 615 nm and exciting at 360 nm: 0.25 eq**

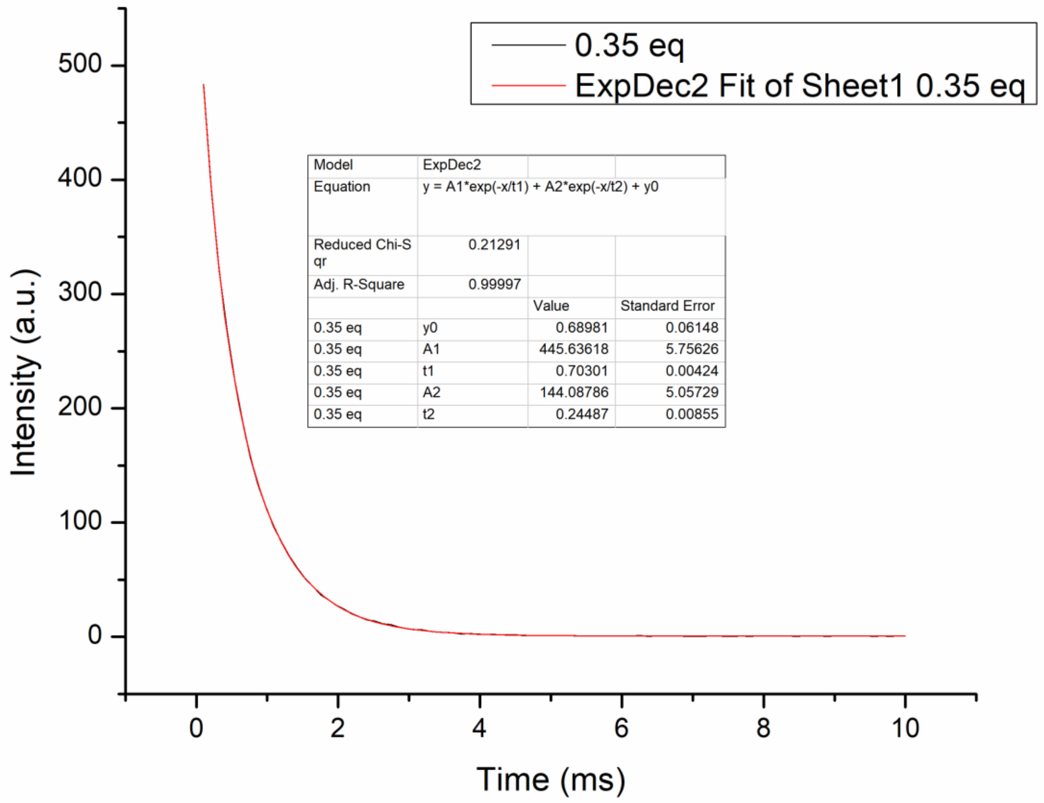
0.35 eq
ExpDec2 Fit of Sheet1 0.35 eq
Model
ExpDec2
Equation
y = A1*exp(-x/t1) + A2*exp(-x/t2) + y0
Reduced Chi-Sqr
0.21291
Adj. R-Square
0.99997
Value
Standard Error
0.35 eq
y0
0.68981
0.06148
A1
445.63618
5.75626
t1
0.70301
0.00424
A2
144.08786
5.05729
t2
0.24487
0.00855
Intensity (a.u.)
Time (ms)
500
400
300
200
100
0
2
4
6
8
10

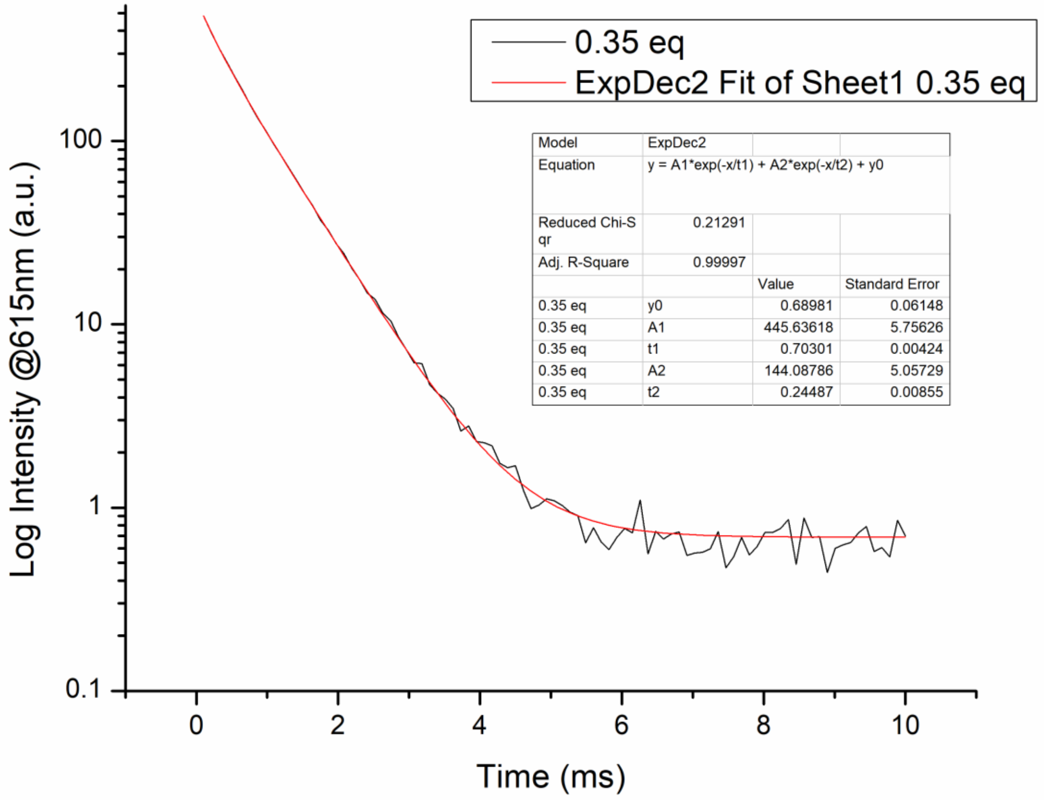


**Figure S 31. Fluorescence decay fitting of $L_1^{2-}$ in Tris HCl with increasing amounts of $Eu^{3+}$ monitoring at 615 nm and exciting at 360 nm: 0.35 eq**

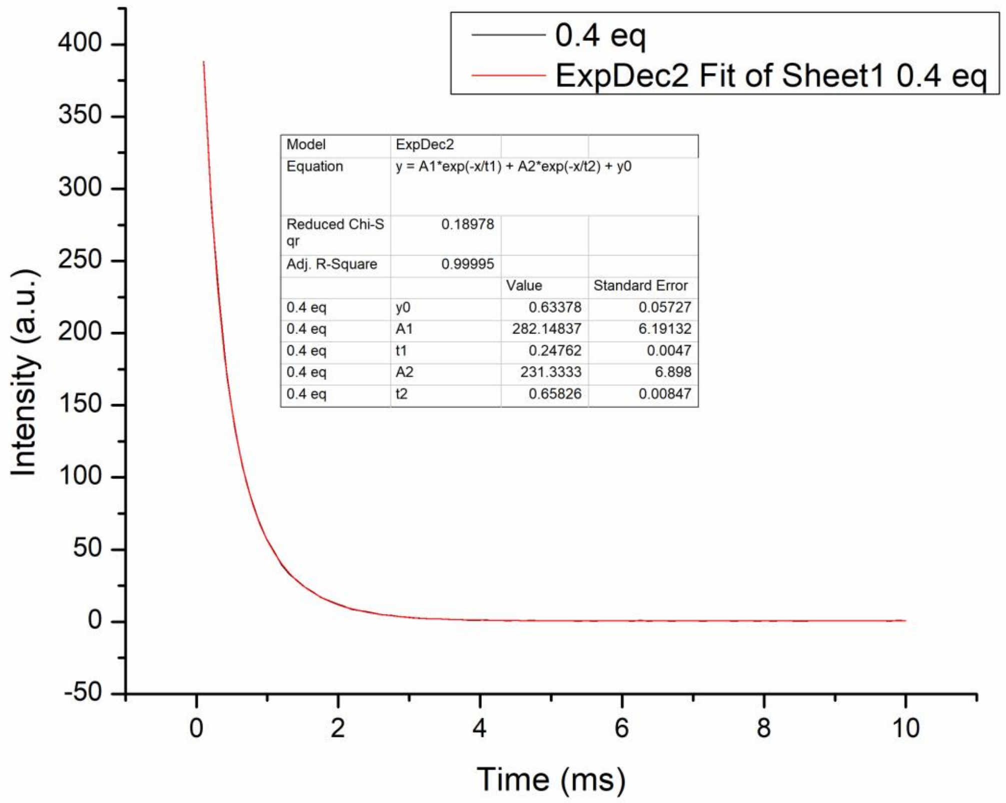
0.4 eq
ExpDec2 Fit of Sheet1 0.4 eq
Model ExpDec2
Equation y = A1*exp(-x/t1) + A2*exp(-x/t2) + y0
Reduced Chi-Sqr 0.18978
Adj. R-Square 0.99995
Value Standard Error
0.4 eq y0 0.63378 0.05727
0.4 eq A1 282.14837 6.19132
0.4 eq t1 0.24762 0.0047
0.4 eq A2 231.3333 6.898
0.4 eq t2 0.65826 0.00847
Intensity (a.u.)
Time (ms)
400
350
300
250
200
150
100
50
0
-50
0
2
4
6
8
10

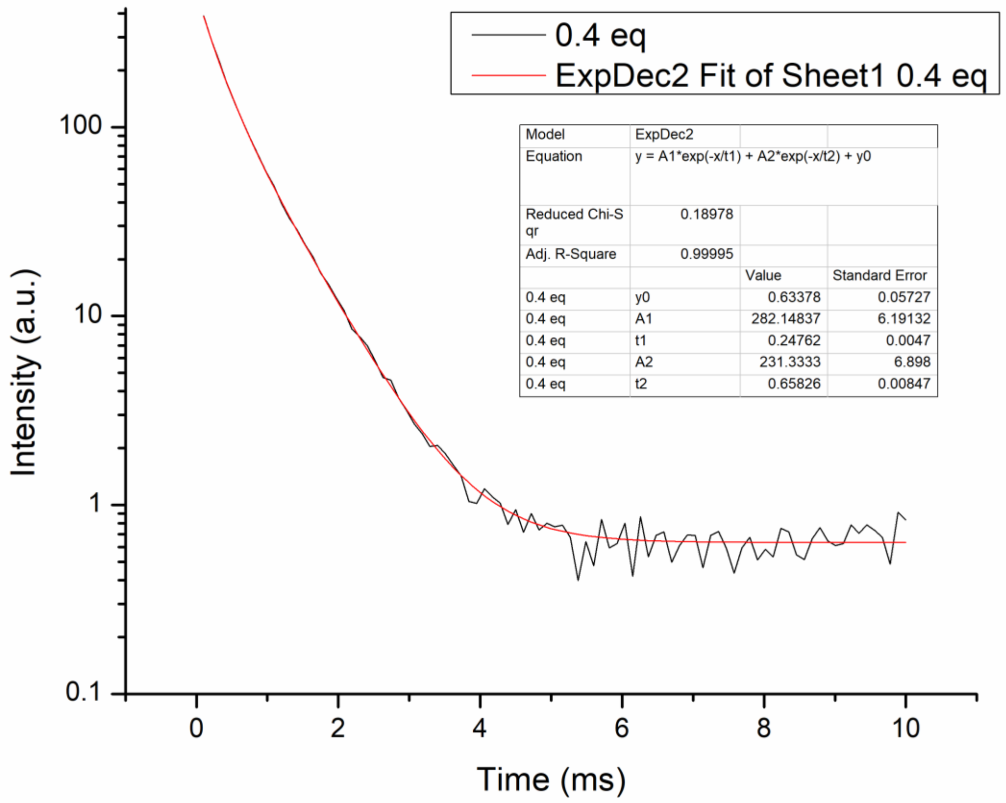


**Figure S 32. Fluorescence decay fitting of $L_1^{2-}$ in Tris HCl with increasing amounts of $Eu^{3+}$ monitoring at 615 nm and exciting at 360 nm: 0.4 eq**

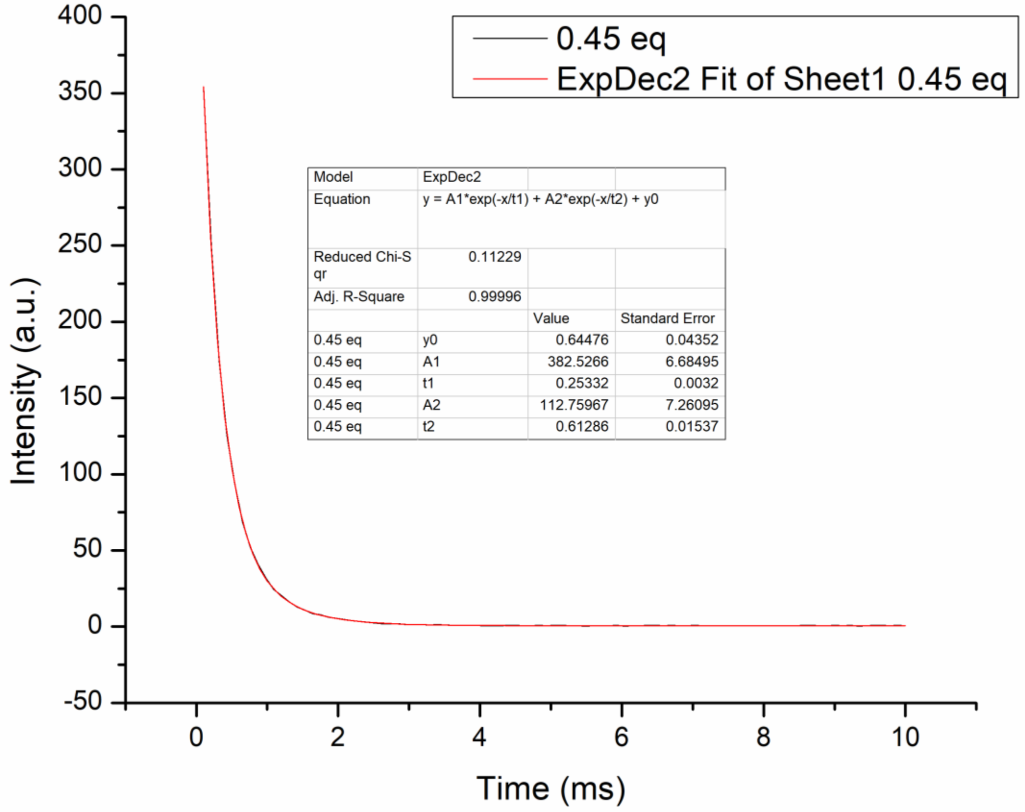
400
350
300
250
200
150
100
50
0
-50
Intensity (a.u.)
0
2
4
6
8
10
Time (ms)
0.45 eq
ExpDec2 Fit of Sheet1 0.45 eq
Model
ExpDec2
Equation
y = A1*exp(-x/t1) + A2*exp(-x/t2) + y0
Reduced Chi-Sqr
0.11229
Adj. R-Square
0.99996
Value
Standard Error
0.45 eq
y0
0.64476
0.04352
0.45 eq
A1
382.5266
6.68495
0.45 eq
t1
0.25332
0.0032
0.45 eq
A2
112.75967
7.26095
0.45 eq
t2
0.61286
0.01537

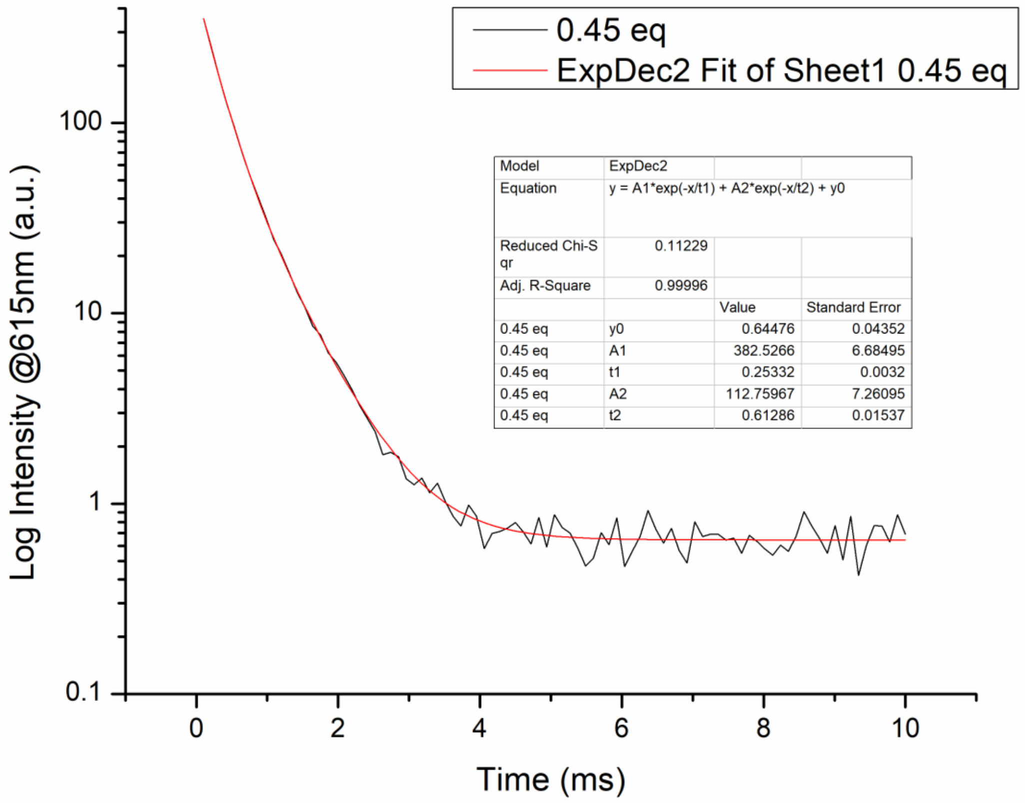


| Model | ExpDec2 | | |
|---|---|---|---|
| Equation | y = A1*exp(-x/t1) + A2*exp(-x/t2) + y0 | | |
| Reduced Chi-Sqr | 0.11229 | | |
| Adj. R-Square | 0.99996 | | |
| | | Value | Standard Error |
| 0.45 eq | y0 | 0.64476 | 0.04352 |
| 0.45 eq | A1 | 382.5266 | 6.68495 |
| 0.45 eq | t1 | 0.25332 | 0.0032 |
| 0.45 eq | A2 | 112.75967 | 7.26095 |
| 0.45 eq | t2 | 0.61286 | 0.01537 |

**Figure S 33. Fluorescence decay fitting of $L_1^{2-}$ in Tris HCl with increasing amounts of $Eu^{3+}$ monitoring at 615 nm and exciting at 360 nm: 0.45 eq**

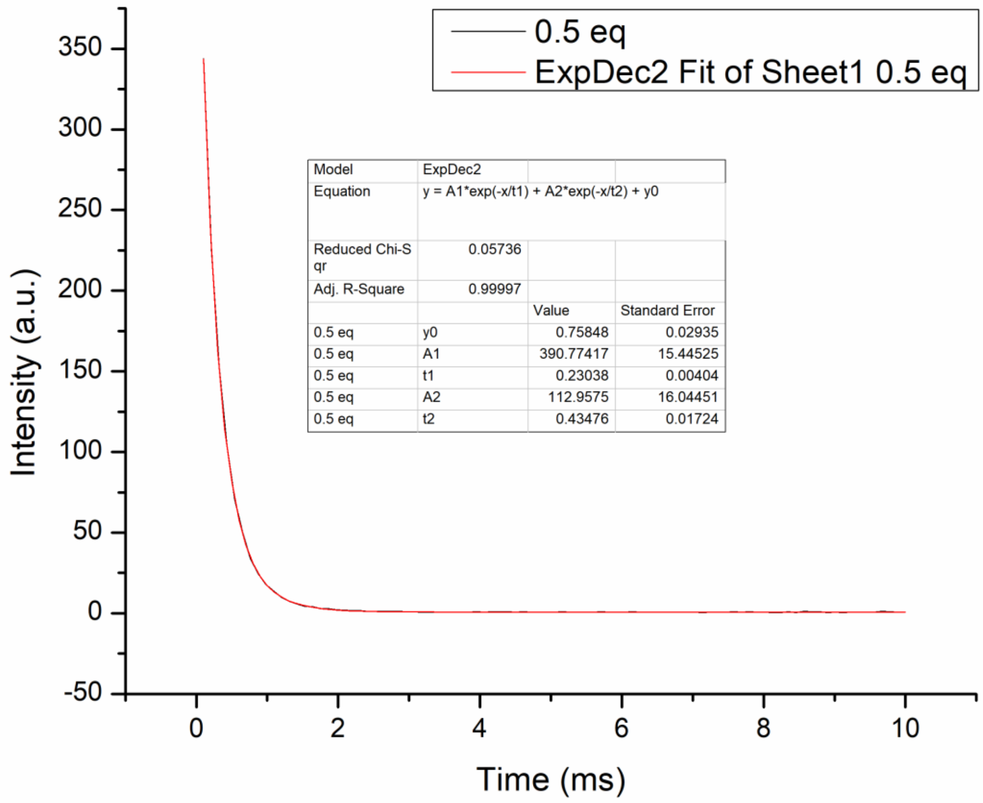


| Model | ExpDec2 | | |
|---|---|---|---|
| Equation | y = A1*exp(-x/t1) + A2*exp(-x/t2) + y0 | | |
| Reduced Chi-Sqr | 0.05736 | | |
| Adj. R-Square | 0.99997 | | |
| | | Value | Standard Error |
| 0.5 eq | y0 | 0.75848 | 0.02935 |
| 0.5 eq | A1 | 390.77417 | 15.44525 |
| 0.5 eq | t1 | 0.23038 | 0.00404 |
| 0.5 eq | A2 | 112.9575 | 16.04451 |
| 0.5 eq | t2 | 0.43476 | 0.01724 |

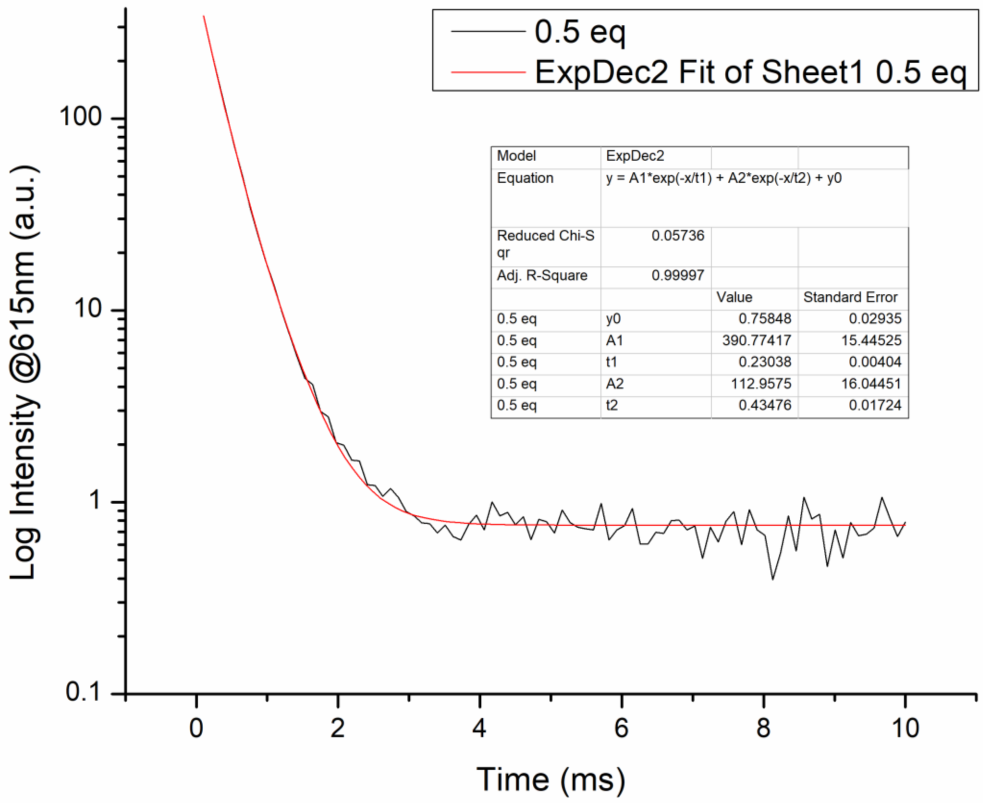


**Figure S 34. Fluorescence decay fitting of $L_1^{2-}$ in Tris HCl with increasing amounts of $Eu^{3+}$ monitoring at 615 nm and exciting at 360 nm: 0.5 eq**

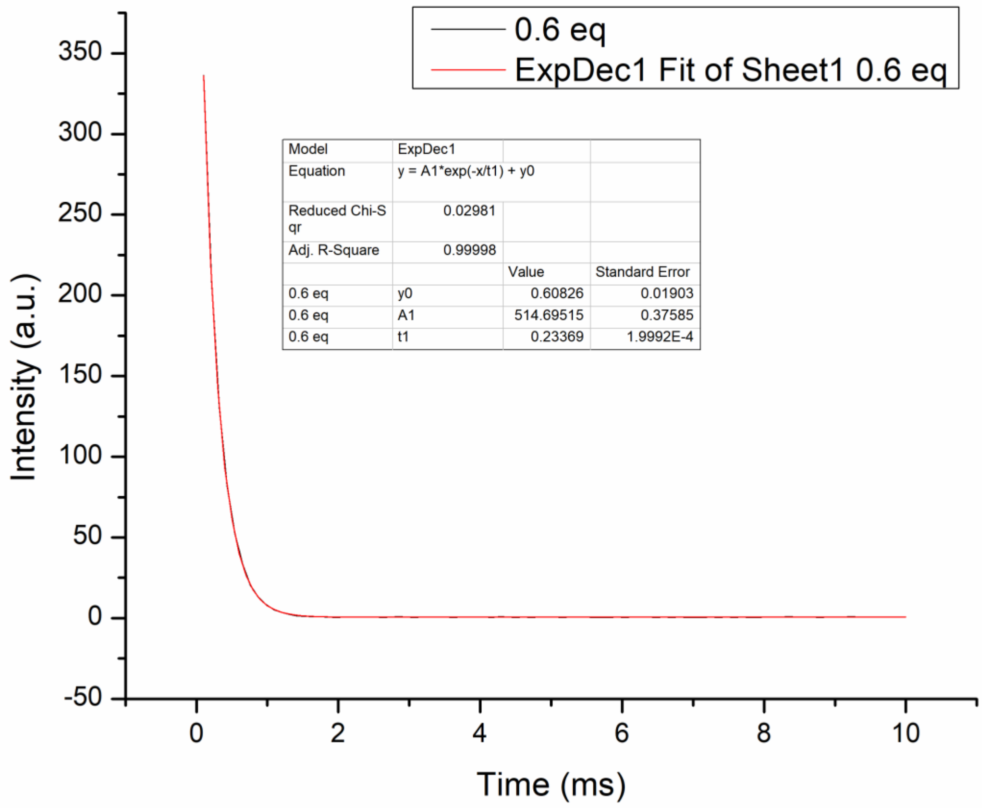
0.6 eq
ExpDec1 Fit of Sheet1 0.6 eq
Model ExpDec1
Equation y = A1*exp(-x/t1) + y0
Reduced Chi-Sqr 0.02981
Adj. R-Square 0.99998
Value Standard Error
0.6 eq y0 0.60826 0.01903
0.6 eq A1 514.69515 0.37585
0.6 eq t1 0.23369 1.9992E-4
Intensity (a.u.)
Time (ms)
350
300
250
200
150
100
50
0
-50
0
2
4
6
8
10

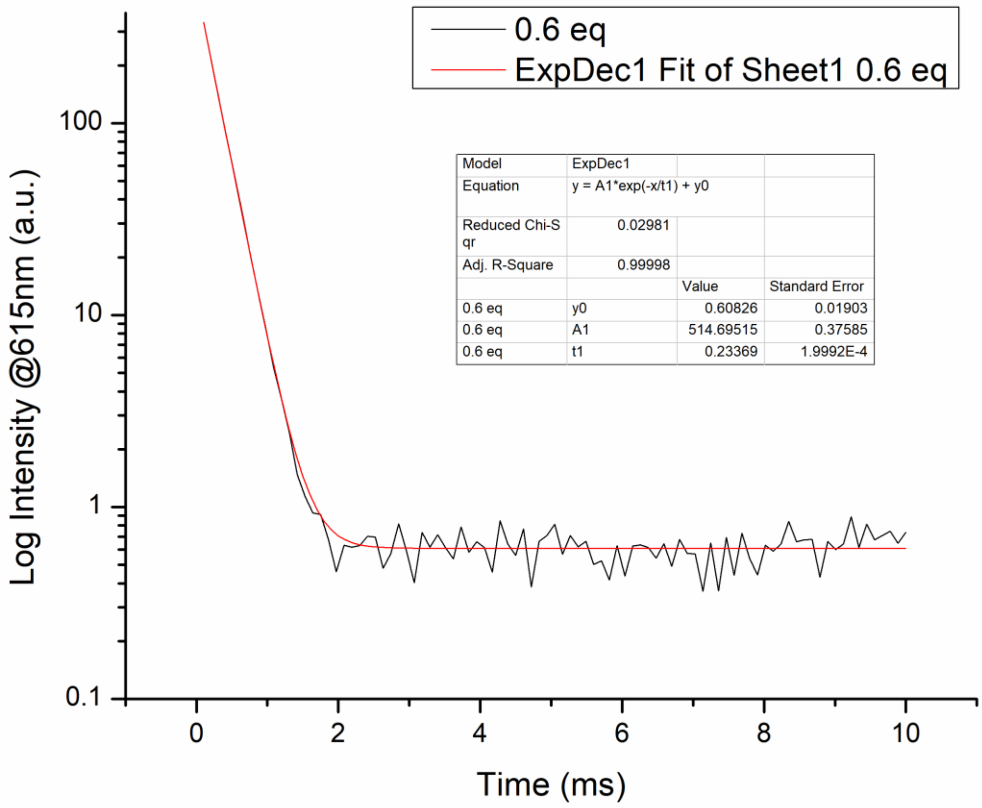


**Figure S 35. Fluorescence decay fitting of $L_1^{2-}$ in Tris HCl with increasing amounts of $Eu^{3+}$ monitoring at 615 nm and exciting at 360 nm: 0.6 eq**

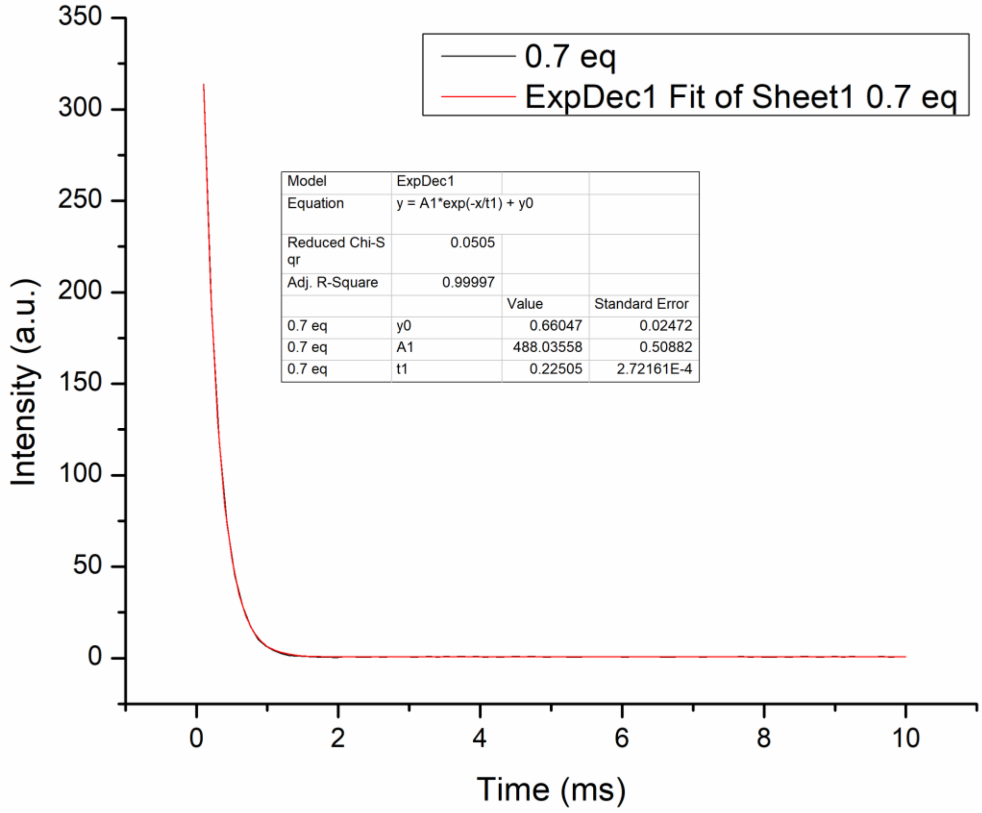
350
300
250
200
150
100
50
0
Intensity (a.u.)
0
2
4
6
8
10
Time (ms)
0.7 eq
ExpDec1 Fit of Sheet1 0.7 eq
Model ExpDec1
Equation y = A1*exp(-x/t1) + y0
Reduced Chi-Sqr 0.0505
Adj. R-Square 0.99997
Value Standard Error
0.7 eq y0 0.66047 0.02472
0.7 eq A1 488.03558 0.50882
0.7 eq t1 0.22505 2.72161E-4

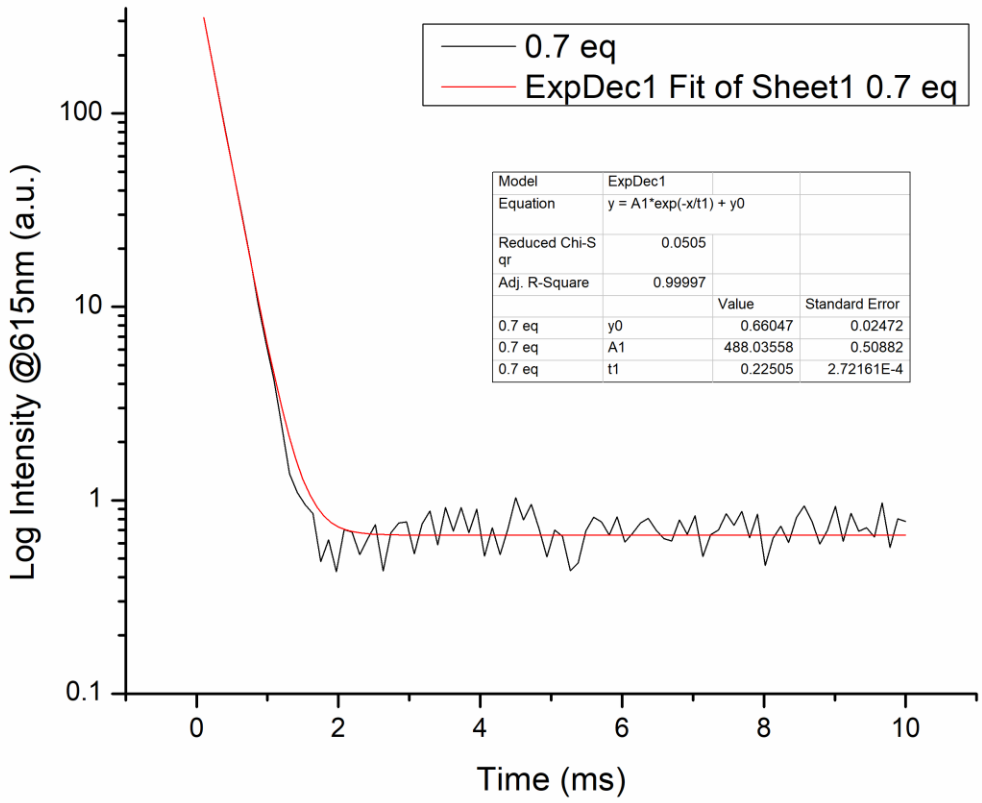


**Figure S 36. Fluorescence decay fitting of $L_1^{2-}$ in Tris HCl with increasing amounts of $Eu^{3+}$ monitoring at 615 nm and exciting at 360 nm: 0.7 eq**

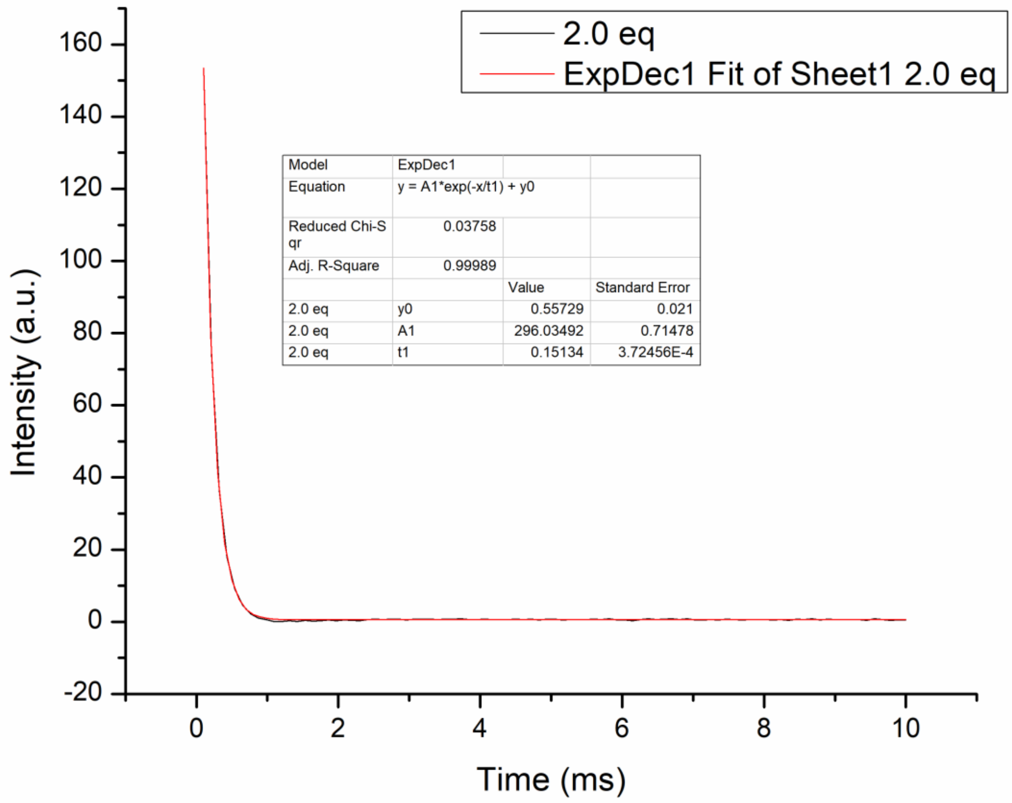
2.0 eq
ExpDec1 Fit of Sheet1 2.0 eq
Model ExpDec1
Equation y = A1*exp(-x/t1) + y0
Reduced Chi-Sqr 0.03758
Adj. R-Square 0.99989
Value Standard Error
2.0 eq y0 0.55729 0.021
2.0 eq A1 296.03492 0.71478
2.0 eq t1 0.15134 3.72456E-4
Intensity (a.u.)
Time (ms)

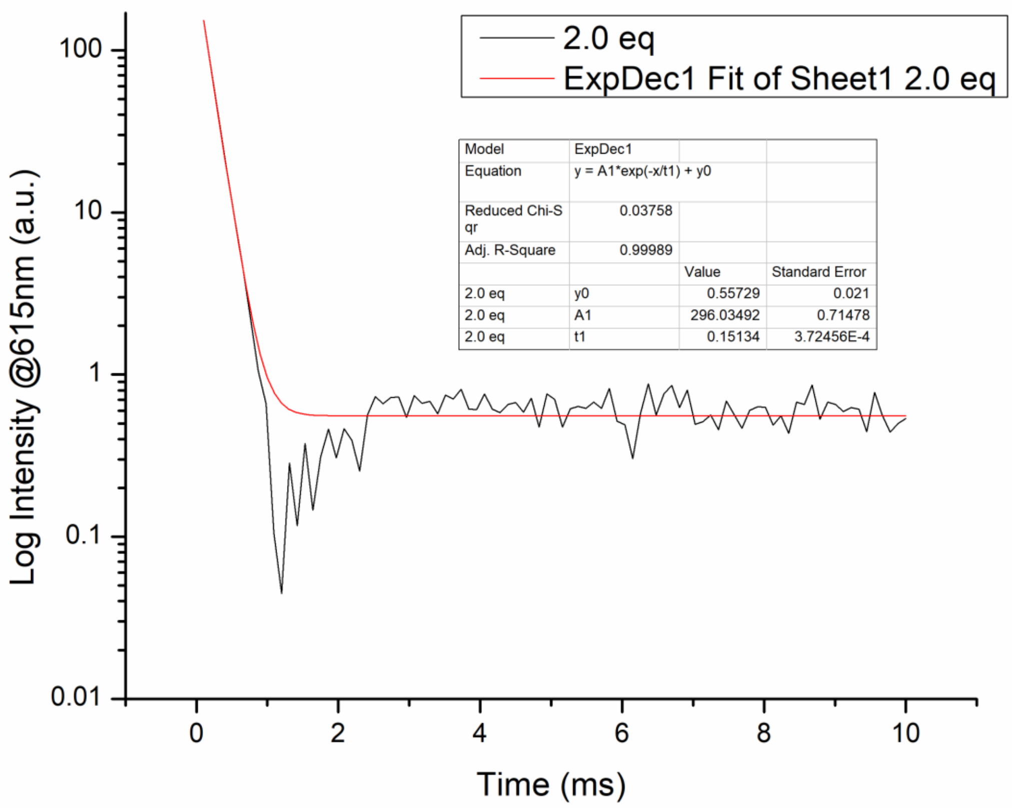


**Figure S 37. Fluorescence decay fitting of $L_1^{2-}$ in Tris HCl with increasing amounts of $Eu^{3+}$ monitoring at 615 nm and exciting at 360 nm: 2 eq**

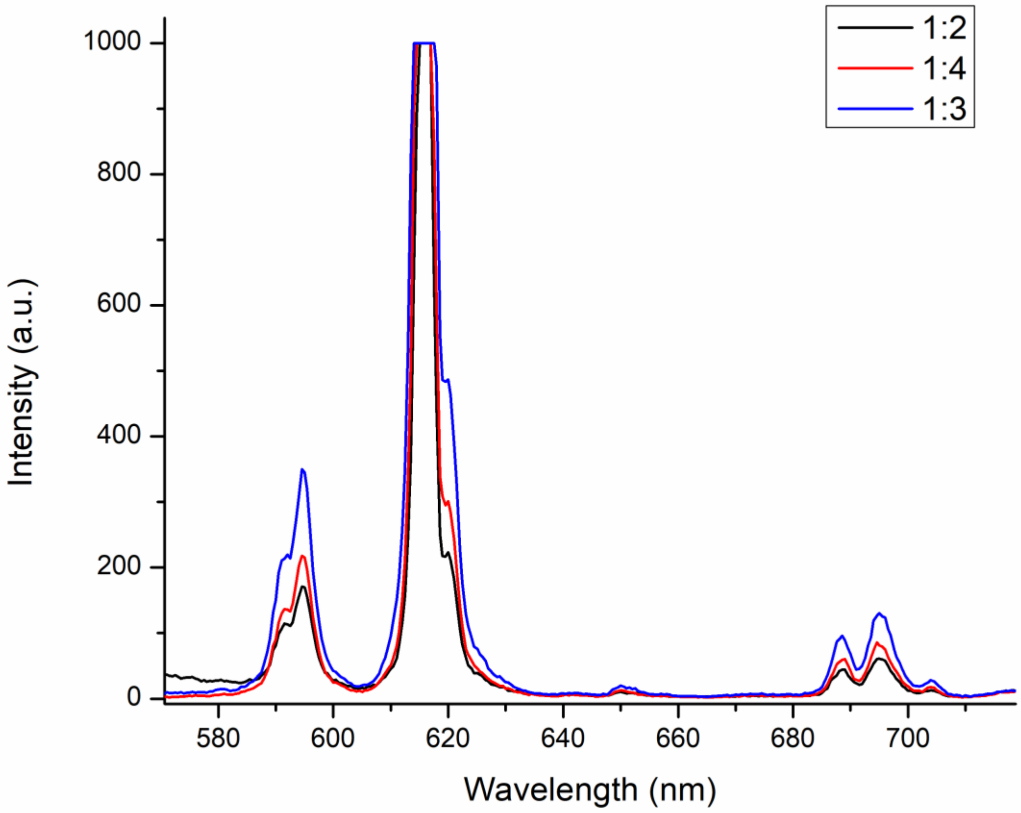


**Figure S 38. Fluorescence spectra comparison (excitation at 360 nm) in DMSO between $Eu^{3+}/L_2^{2-}$ in 1:2 (black), 1:3 (blue) and 1:4 (red) molar ratios.**

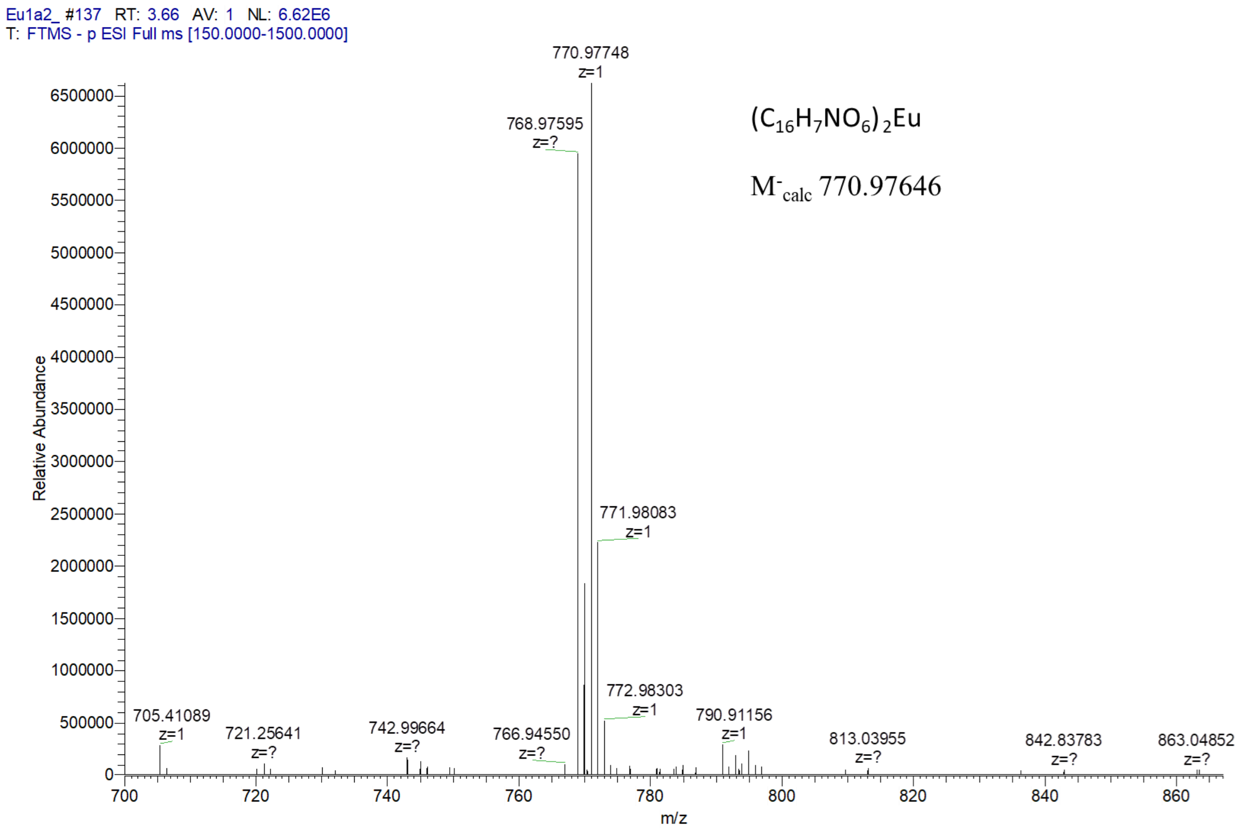
Eu1a2_ #137 RT: 3.66 AV: 1 NL: 6.62E6
T: FTMS - p ESI Full ms [150.0000-1500.0000]
770.97748
z=1
768.97595
z=?
771.98083
z=1
772.98303
z=1
705.41089
z=1
721.25641
z=?
742.99664
z=?
766.94550
z=?
790.91156
z=1
813.03955
z=?
842.83783
z=?
863.04852
z=?
Relative Abundance
m/z
(C16H7NO6)2Eu
M-calc 770.97646

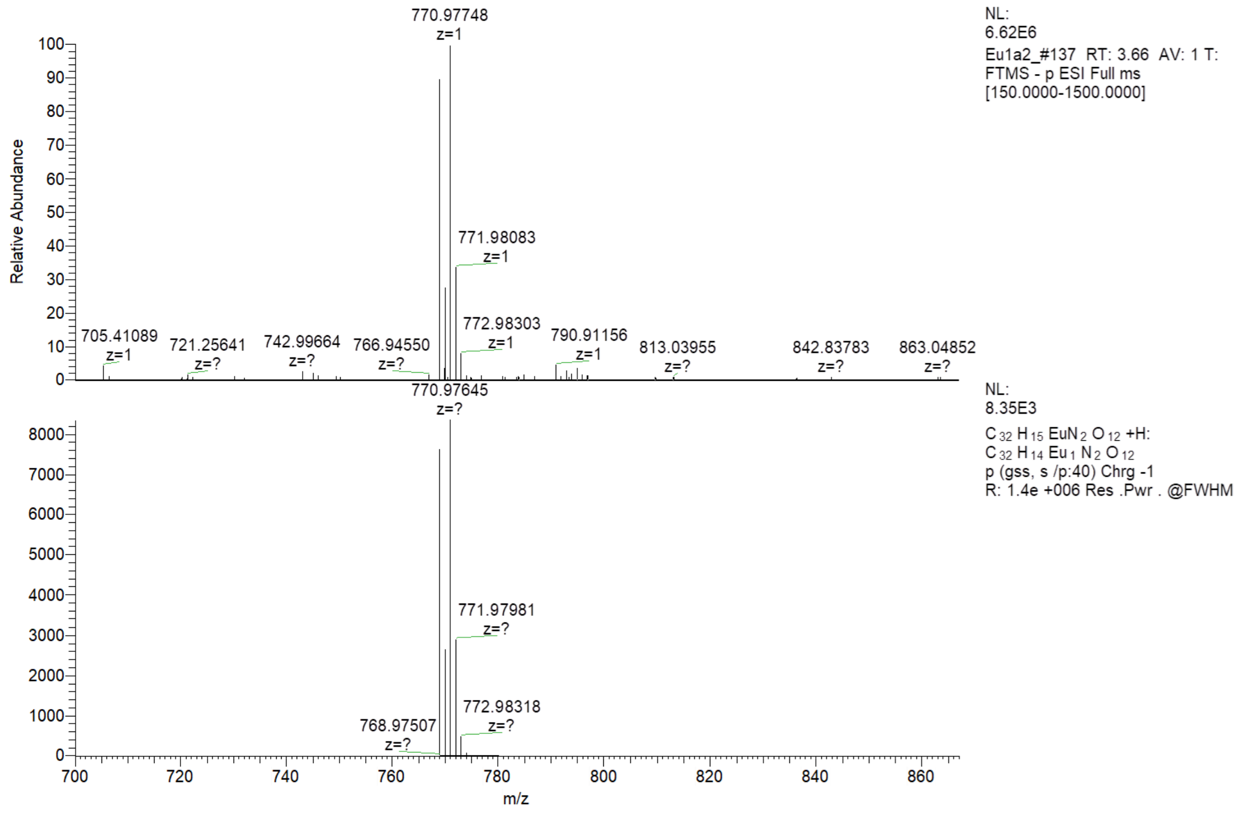


**Figure S 39. HRMS spectra of DMSO mixture $Eu^{3+}$:$L_1^{2-}$ in 1:2 molar ratio.**

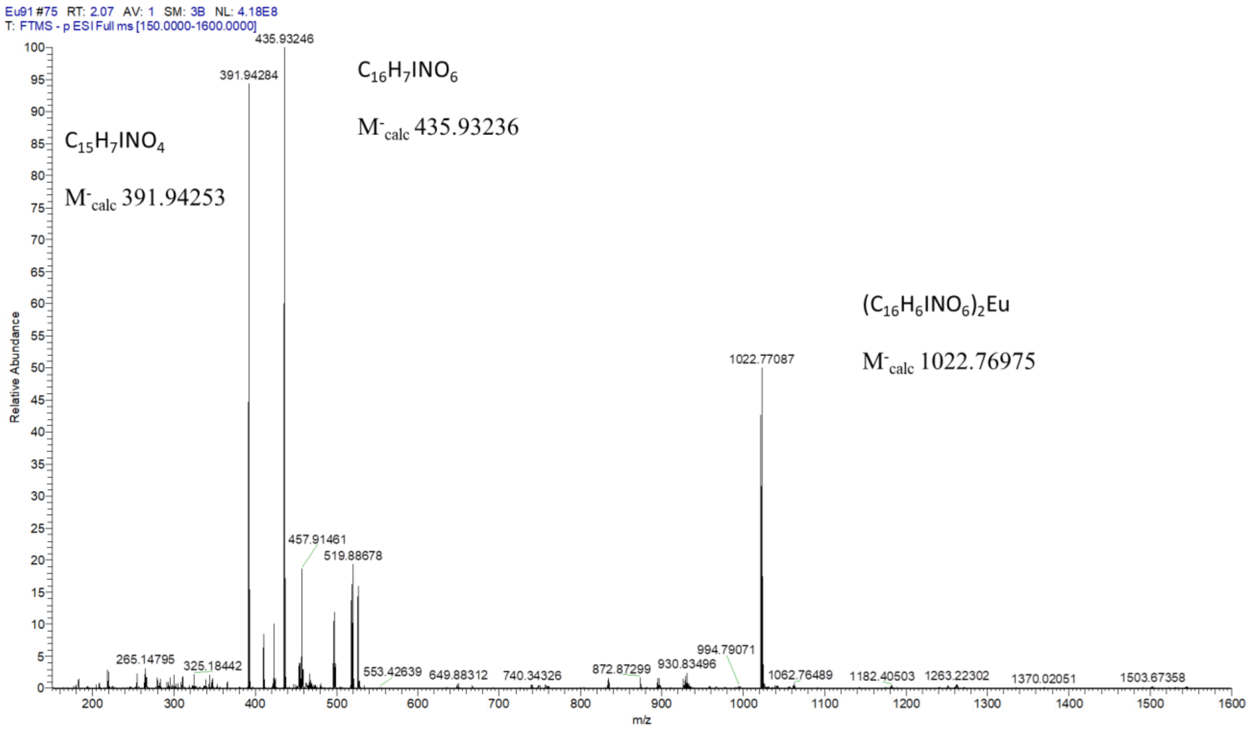


**Figure S 40. HMRS spectrum of DMSO mixture $Eu^{3+}$:$L_2^{2-}$ in 1:2 molar ratio.**

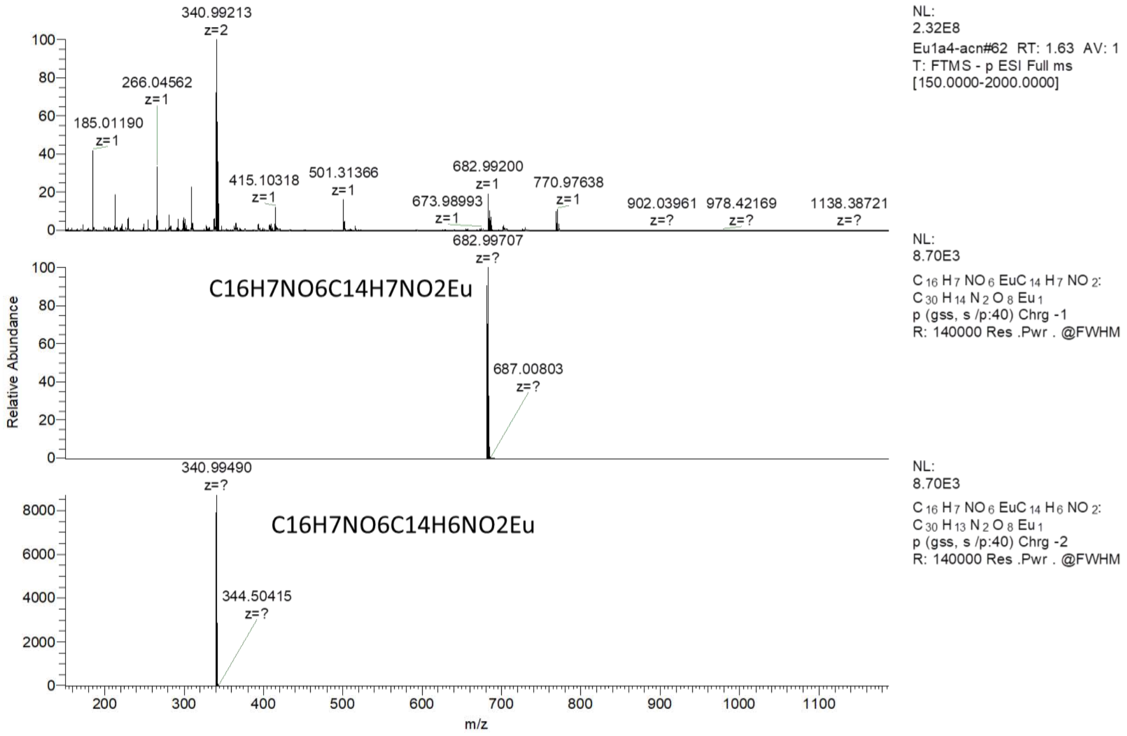


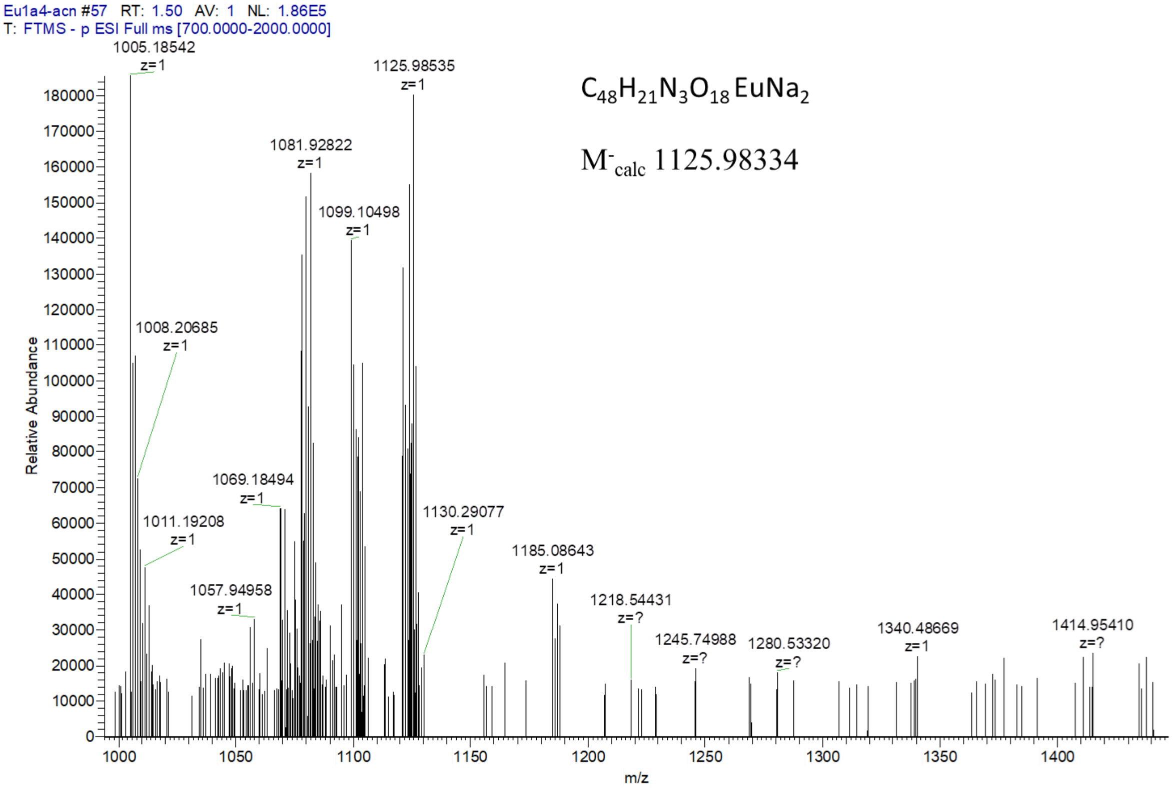


**Figure S 41. HMRS spectrum of DMSO mixture $Eu^{3+}$:$L_1^{2-}$ in 1:4 molar ratio.**

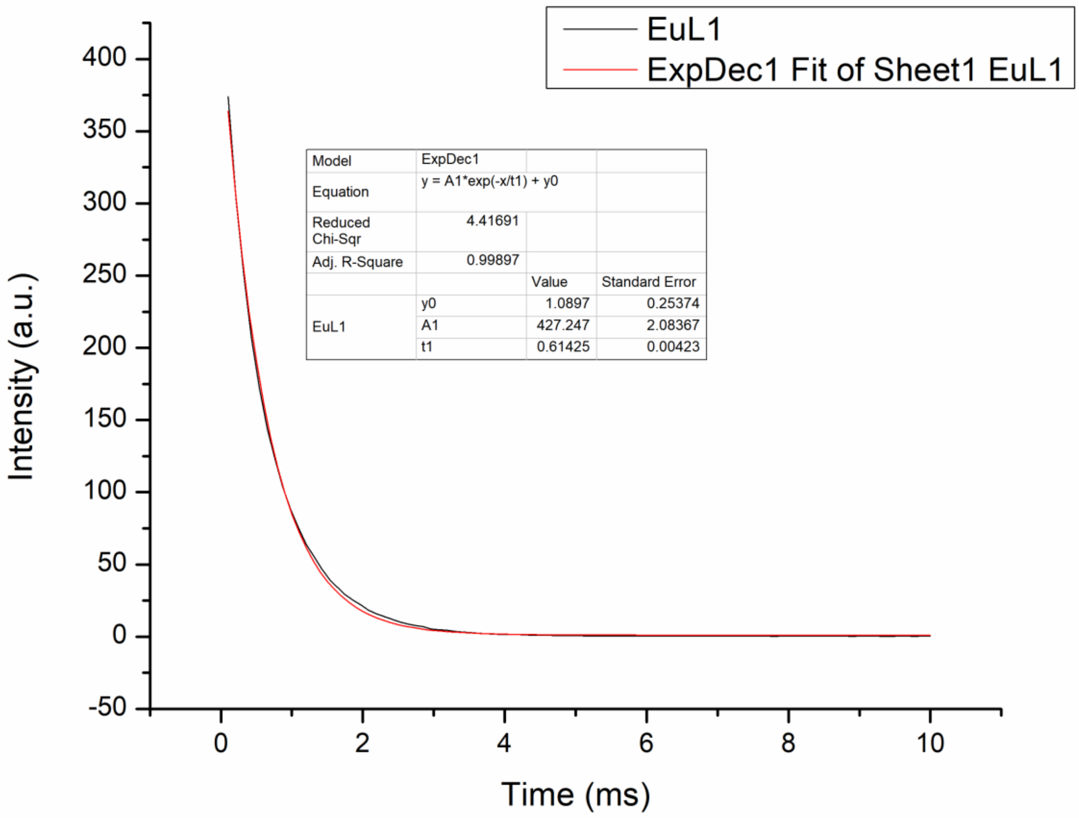
EuL1
ExpDec1 Fit of Sheet1 EuL1
Model
ExpDec1
Equation
y = A1*exp(-x/t1) + y0
Reduced Chi-Sqr
4.41691
Adj. R-Square
0.99897
Value
Standard Error
EuL1
y0
1.0897
0.25374
A1
427.247
2.08367
t1
0.61425
0.00423
Intensity (a.u.)
Time (ms)
400
350
300
250
200
150
100
50
0
-50
0
2
4
6
8
10

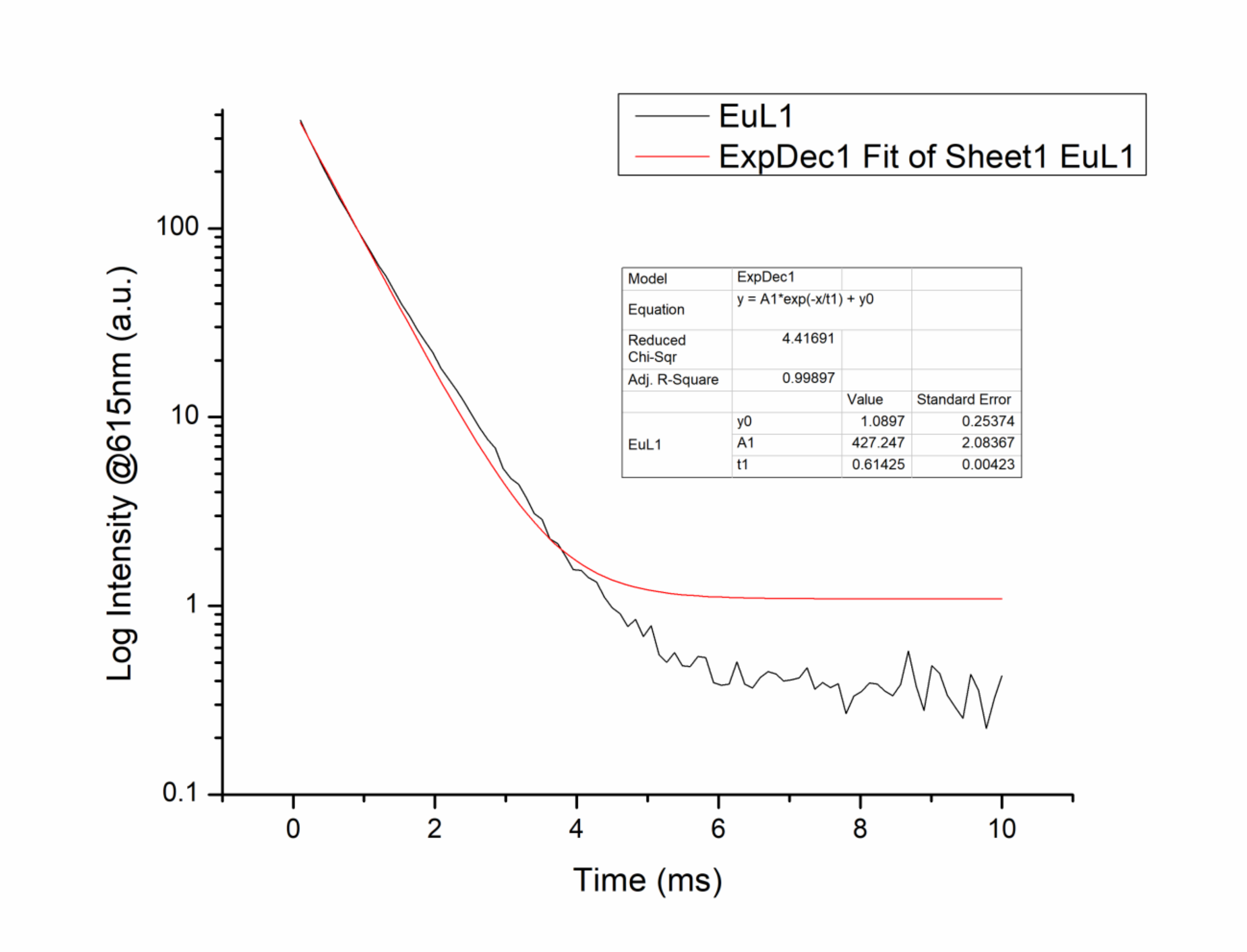


**Figure S 42. Fluorescence decay fitting of isolated $Na_3[Eu(L_1)_3]$ in water, monitoring at 615 nm and exciting at 360 nm.**

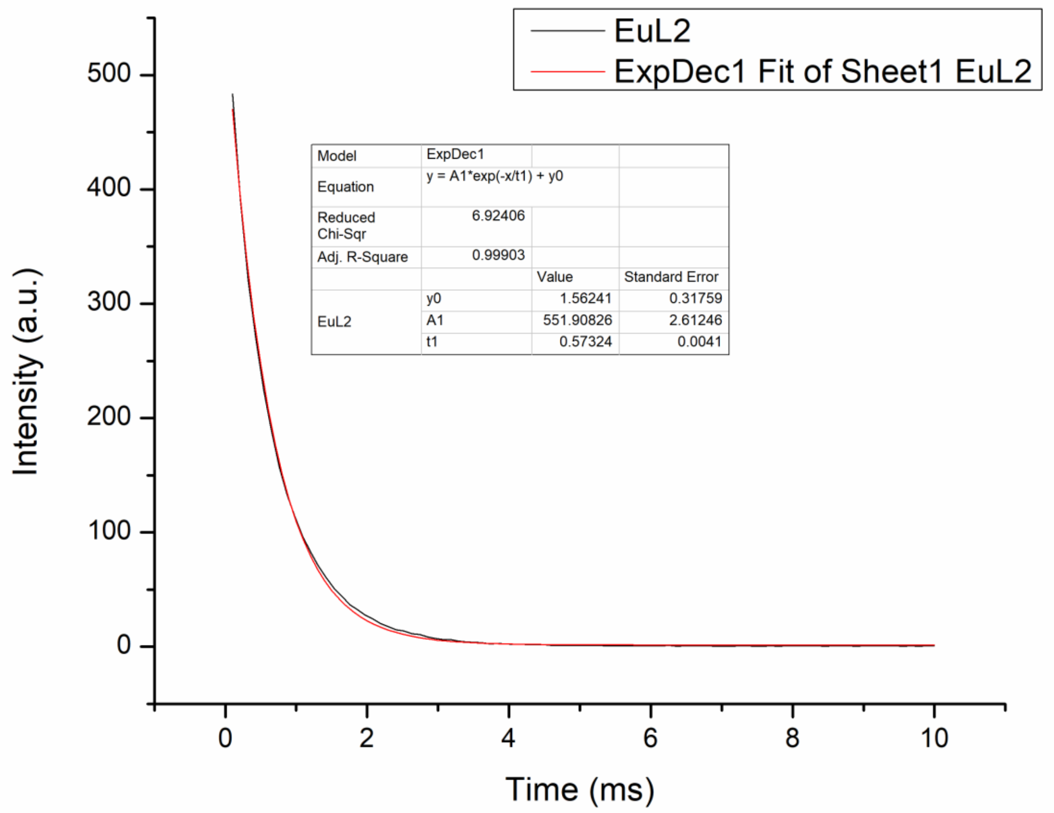
EuL2
ExpDec1 Fit of Sheet1 EuL2
Model
ExpDec1
Equation
y = A1*exp(-x/t1) + y0
Reduced Chi-Sqr
6.92406
Adj. R-Square
0.99903
Value
Standard Error
EuL2
y0
1.56241
0.31759
A1
551.90826
2.61246
t1
0.57324
0.0041
Intensity (a.u.)
Time (ms)

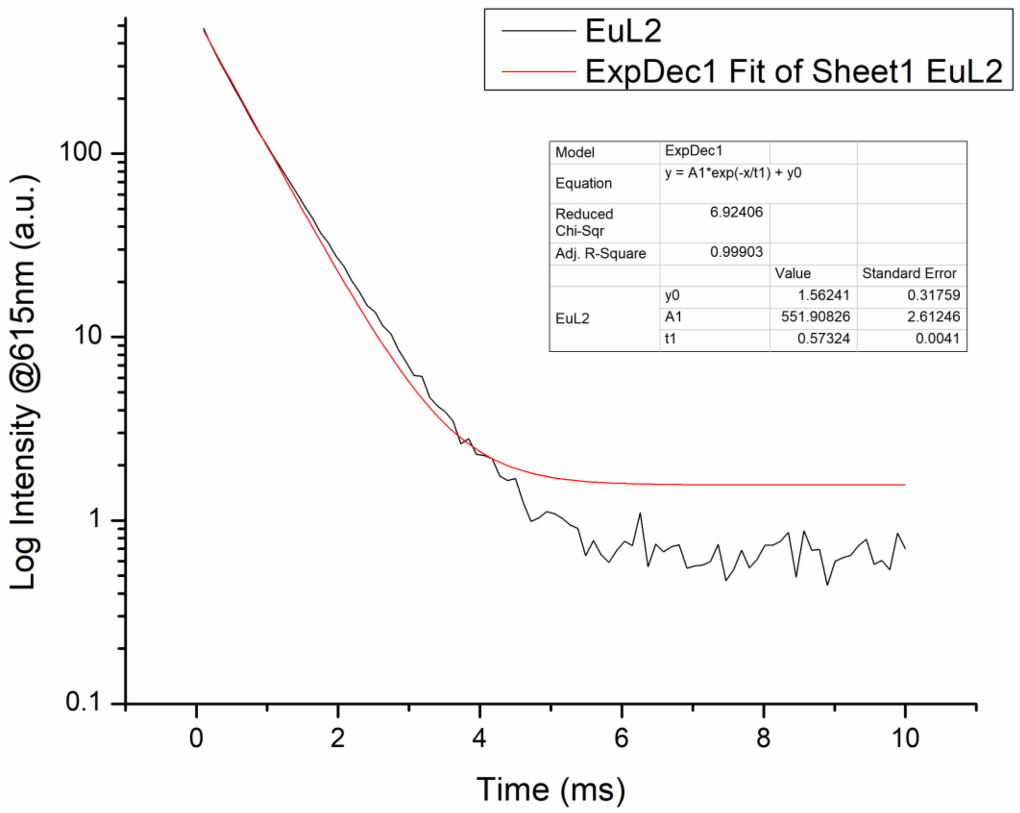


**Figure S 43. Fluorescence decay fitting of isolated $Na_3[Eu(L_2)_3]$ in water, monitoring at 615 nm and exciting at 360 nm.**

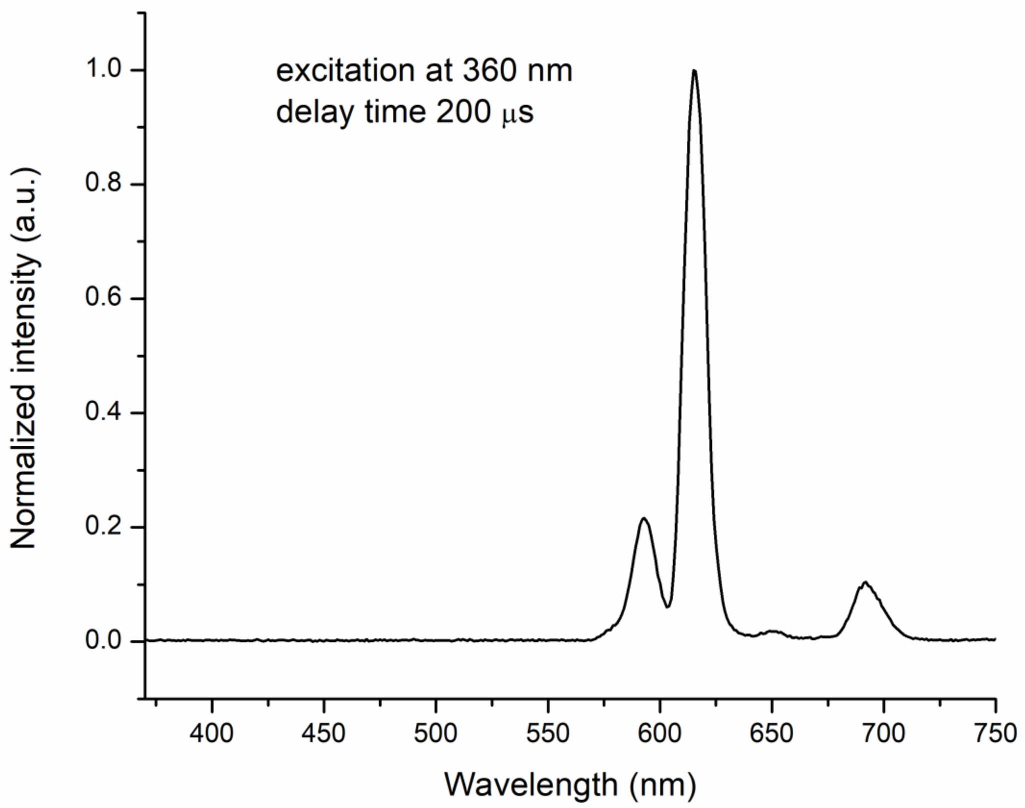


**Figure S 44. Phosphorescence spectrum of $Na_3[Eu(L_1)_3]$ complex in water with 200μs of delay time.**

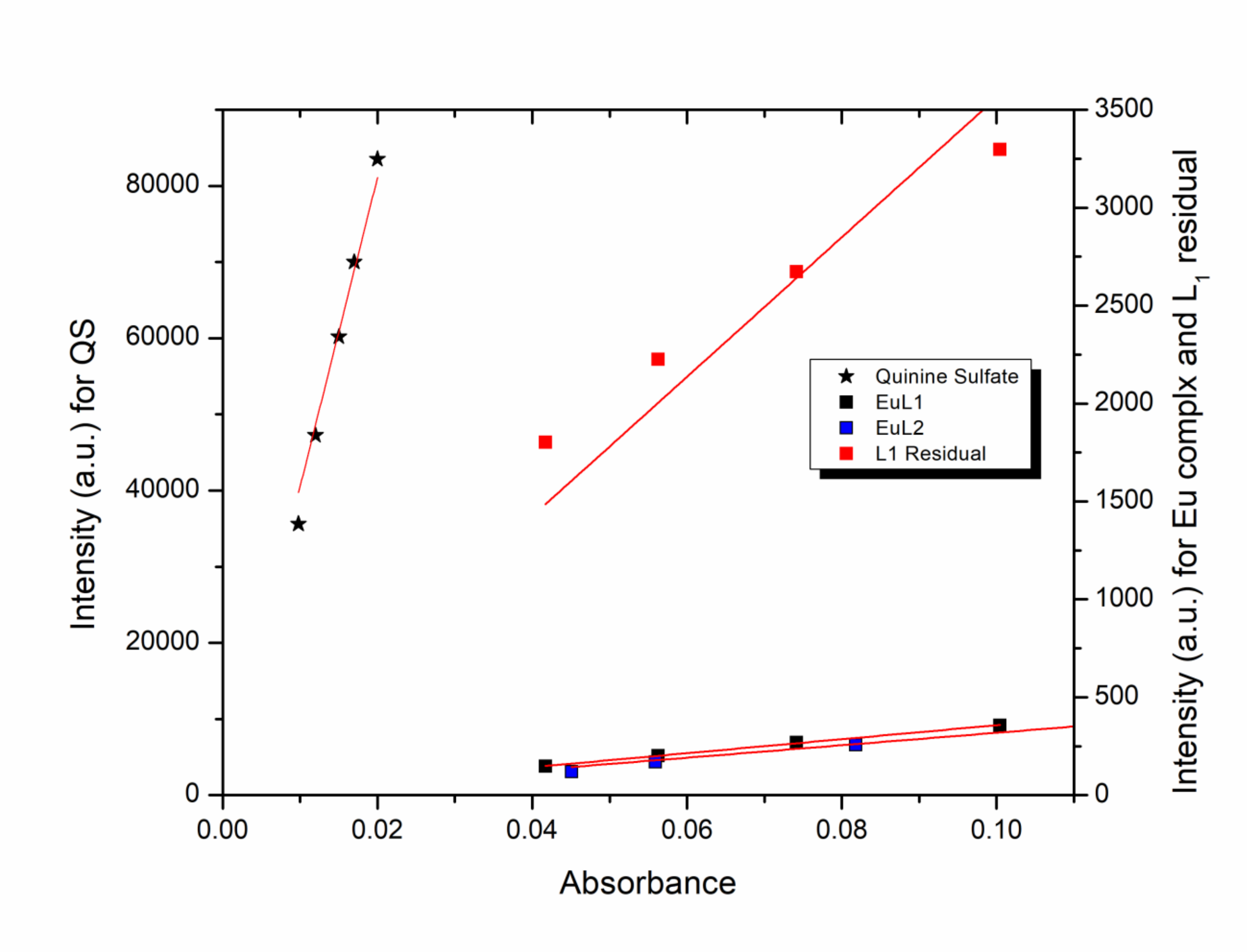


| | A | B | C | D |
|---|---|---|---|---|
| 1 | Equation | y = a + b*x | | |
| 2 | Weight | No Weighting | | |
| 3 | Residual Sum of Squares | 25.31818 | 858.61091 | 228267.4124 |
| 4 | Pearson's r | 0.99995 | 0.9983 | 0.99564 |
| 5 | Adj. R-Square | 0.99987 | 0.99547 | 0.98839 |
| 6 | | | Value | Standard Error |
| 7 | EuL1 | Intercept | 0 | -- |
| 8 | | Slope | 3573.43801 | 20.29393 |
| 9 | EuL2 | Intercept | 0 | -- |
| 10 | | Slope | 3188.71511 | 107.44075 |
| 11 | L1 Residual | Intercept | 0 | -- |
| 12 | | Slope | 35618.79804 | 1926.95606 |

**Figure S 45. Quantum yields plot of isolated tris complexes in water solution (above) and fitting details (below).**

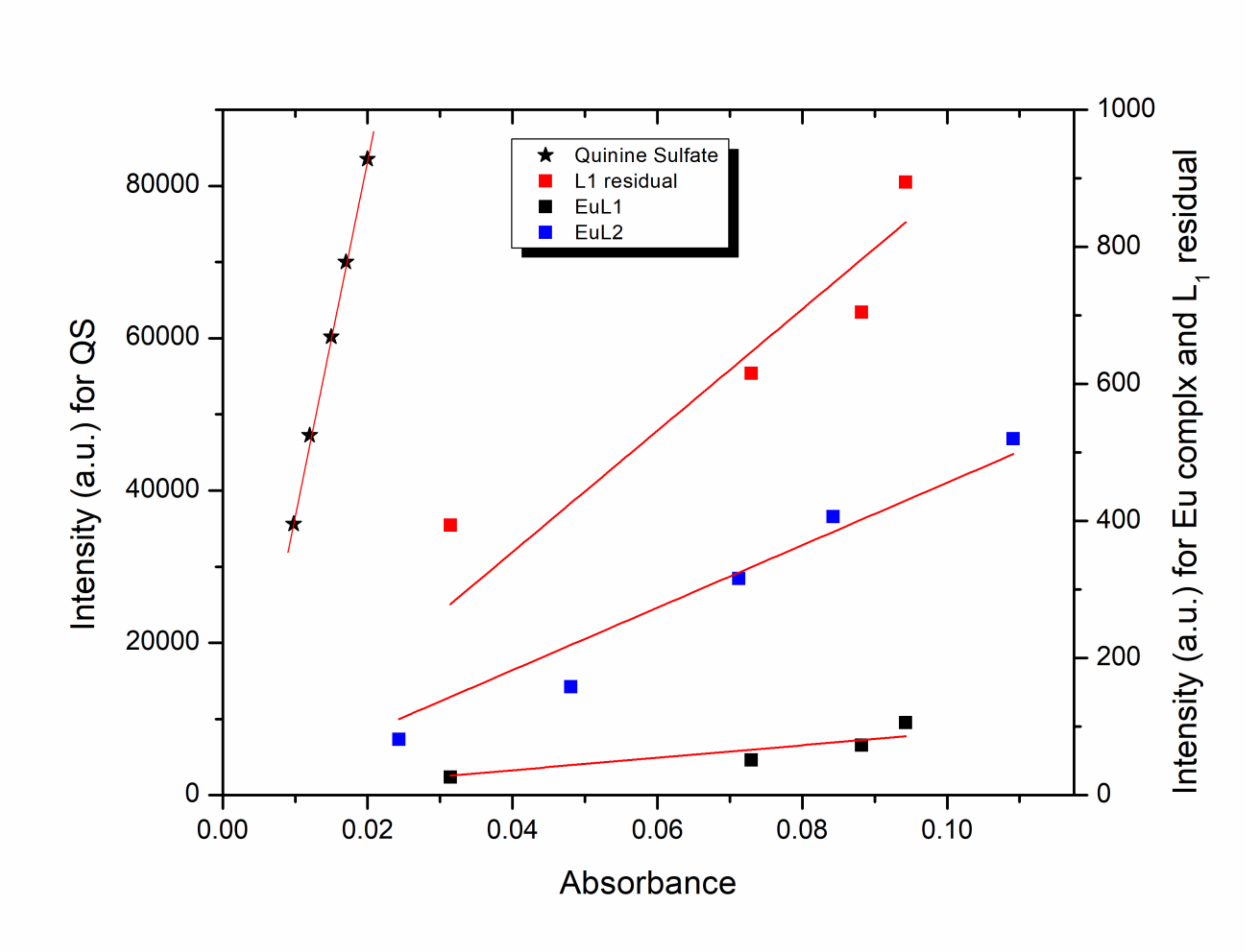


| | A | B | C | D |
|---|---|---|---|---|
| 1 | Equation | y = a + b*x | | |
| 2 | Weight | No Weighting | | |
| 3 | Residual Sum of Squares | 23754.20021 | 682.19915 | 5694.37765 |
| 4 | Pearson's r | 0.99349 | 0.98258 | 0.99497 |
| 5 | Adj. R-Square | 0.98269 | 0.95394 | 0.98744 |
| 6 | | | Value | Standard Error |
| 7 | L1 residual | Intercept | 0 | -- |
| 8 | | Slope | 8865.68405 | 587.03511 |
| 9 | EuL1 | Intercept | 0 | -- |
| 10 | | Slope | 910.95335 | 99.48313 |
| 11 | EuL2 | Intercept | 0 | -- |
| 12 | | Slope | 4560.45093 | 229.68249 |

**Figure S 46. Quantum yields plot of isolated tris complexes in DMSO solution (above) and fitting details (below).**

| | A | B | C | D |
|---|---|---|---|---|
| 1 | Equation | y = a + b*x | | |
| 2 | Weight | No Weighting | | |
| 3 | Residual Sum of Squares | 2.62986E7 | | |
| 4 | Pearson's r | 0.99931 | | |
| 5 | Adj. R-Square | 0.99827 | | |
| 6 | | | Value | Standard Error |
| 7 | Quinine Sulfate | Intercept | 0 | -- |
| 8 | | Slope | 4.05511E6 | 75478.94658 |

**Figure S 47. Fitting details of quinine sulfate in 0.5 M $H_2SO_4$.**
